# The E3 ubiquitin ligase Nedd4 fosters developmental myelination in the mouse central and peripheral nervous system

**DOI:** 10.1002/glia.24642

**Published:** 2024-11-07

**Authors:** Cristina Fimiani, Jorge A. Pereira, Joanne Gerber, Ingrid Berg, Jonathan DeGeer, Sven Bachofner, Jonas S. Fischer, Manuel Kauffmann, Ueli Suter

**Affiliations:** ^1^ Institute of Molecular Health Sciences Department of Biology, ETH Zurich Zurich Switzerland

**Keywords:** myelination, Nedd4, oligodendrocytes, radial sorting, Schwann cells

## Abstract

Ubiquitination is a major post‐translational regulatory mechanism that tunes numerous aspects of ubiquitinated target proteins, including localization, stability, and function. During differentiation and myelination, Oligodendrocytes (OLs) in the central nervous system and Schwann cells (SCs) in the peripheral nervous system undergo major cellular changes, including the tightly controlled production of large and accurate amounts of proteins and lipids. Such processes have been implied to depend on regulatory aspects of ubiquitination, with E3 ubiquitin ligases being generally involved in the selection of specific ubiquitination substrates by ligating ubiquitin to targets and granting target selectivity. In this study, we have used multiple transgenic mouse lines to investigate the functional impact of the E3 ubiquitin ligase Nedd4 in the OL‐ and SC‐lineages. Our findings in the developing spinal cord indicate that Nedd4 is required for the correct accumulation of differentiated OLs and ensures proper myelination, supporting and further expanding previously suggested conceptual models. In sciatic nerves, we found that Nedd4 is required for timely radial sorting of axons by SCs as a pre‐requirement for correct onset of myelination. Moreover, Nedd4 ensures correct myelin thickness in both SCs and spinal cord OLs.

## Main Points

- Developing spinal cord: Nedd4 is required for accurate accumulation of differentiated oligodendrocytes and proper myelination.
- Developing sciatic nerve: Nedd4 is required for correct Schwann cell numbers, timely radial sorting, and proper myelination.

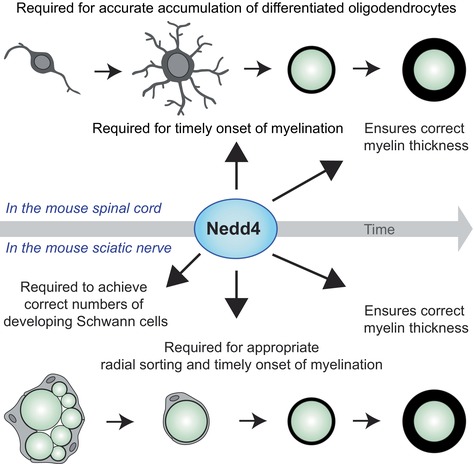

## 1 INTRODUCTION

In jawed vertebrates, oligodendrocytes (OLs) and Schwann cells (SCs) are the myelinating glial cells of the central nervous system (CNS) and peripheral nervous system (PNS), respectively. In the developing OL lineage, OL differentiation involves the conversion of oligodendrocyte precursor cells (OPCs) into OLs and further maturation to enable myelination (Adams et al., 2021; Nave & Werner, 2014). In the PNS, the SC lineage derives from neural crest cells and proceeds through several differentiation stages before SCs start to myelinate axons (Jessen et al., 2015). Prior to and as a prerequisite for myelination, immature SCs sort single large‐caliber axons out of axon bundles to engage in a 1:1 relationship through mechanisms that require expansion of SC numbers and SC process extensions (Feltri et al., 2016).

Myelin consists of massively expanded and specialized cell membrane which surrounds axonal segments in multiple compacted spiral wraps, facilitating rapid action potential propagation (Nave & Werner, 2014). To form and establish myelin in appropriate proportions to the associated axons, OLs and SCs must synthesize large and accurate amounts of proteins and lipids in a timely controlled manner (Bradl & Lassmann, 2010; Chrast et al., 2011; Figlia et al., 2018). Multiple mechanisms are required to ensure the proper regulation involved and homeostasis, including control of adequate protein production and quality control (Figlia et al., 2018; Volpi et al., 2017; Yang et al., 2021). Disturbances in such processes may result in dysfunctional OLs and SCs, contributing to CNS and PNS disorders (Chrast et al., 2011; Nave & Werner, 2014).

Post‐translational protein ubiquitination establishes a complex ubiquitin (Ub) code that can modulate the fate of the ubiquitinated target proteins by inducing changes to features such as stability, protein interactions, localization, and activity (Rape, 2018). In this context, the Ub‐proteasome system (UPS) is involved in modulating levels of functionally critical proteins and in mediating degradation of damaged proteins (Marques et al., 2006; Oh et al., 2018). E3 Ub ligases carry out the transfer of Ub to internal amino acid residues (usually lysines) of specific target proteins. In this way, individual E3 ligases are crucial in determining substrate specificity (Rape, 2018). Dysfunctions in protein ubiquitination and UPS have been implicated in various human diseases, including cancer, autoimmunity, and neurodegeneration (Gómez‐Martín et al., 2008; Schmidt et al., 2021; Sun et al., 2020), emphasizing the importance of Ub‐mediated processes to protect health and homeostasis.

Neural precursor cell expressed, developmentally downregulated 4 (Nedd4, also known as Nedd4‐1) is a E3 Ub ligase (Boase & Kumar, 2015) which plays key roles at multiple stages of neural development (Christie et al., 2012; Drinjakovic et al., 2010; Hsia et al., 2014; Kawabe et al., 2010; Liu et al., 2009; Wiszniak et al., 2013). Beyond development, Nedd4 protects neurons from toxicity by ubiquitinating alpha synuclein and routing it for lysosomal degradation (Davies et al., 2014; Tofaris et al., 2011). In this context, loss of Nedd4 has been correlated with neurodegeneration in Parkinson's disease (Canal et al., 2016; Tofaris et al., 2011). Initial transcriptomics data have revealed that *Nedd4* is expressed by various cell types of the mouse cerebral cortex, including neurons, endothelial cells, astrocytes, and throughout the OL lineage (Zhang et al., 2014). Moreover, single‐cell RNA sequencing has confirmed *Nedd4* expression in the OL lineage (Marques et al., 2016). Proteomics data showed that Nedd4 protein can be detected in the adult mouse brain, in OL‐lineage cells acutely isolated from mouse pups, and in primary OL‐lineage cell cultures obtained from newborn mice (Sharma et al., 2015). Furthermore, Nedd4 expression was found in the OL lineage by analyzing mouse spinal cord and brain tissue sections (Ding et al., 2020). In the mouse PNS, bulk and single‐cell RNA sequencing data indicate that *Nedd4* is widely expressed in the SC lineage (Gerber et al., 2021; Yim et al., 2022). Functionally, it has been demonstrated that Nedd4 promotes neural crest cell survival and regulates aspects of craniofacial and PNS development (Lohraseb et al., 2022; Wiszniak et al., 2013).

Despite constituting different cell types, SCs and OLs share, besides enabling myelination, other common functions including the support and organization of associated axons (Bouçanova & Chrast, 2020; Pease‐Raissi & Chan, 2021; Rasband & Peles, 2021; Stassart et al., 2018). Being a general regulatory mechanism, ubiquitination is likely to play essential roles in both types of myelinating glia. In support of this hypothesis, several E3 ubiquitin ligases have been implicated in the regulation of myelination carried out by SCs and/or by OLs, including Fbxw7 (Harty et al., 2019; Kearns et al., 2015), Herc1 (Bachiller et al., 2018), and Mdm2 (Fumagalli et al., 2015). In our study, we aimed at evaluating the relevance of Nedd4 in the OL lineage and SC lineage during developmental CNS and PNS myelination, respectively. Towards this goal, we used multiple transgenic mouse lines to deplete Nedd4 from each of these lineages. Our results in the developing spinal cord indicate that Nedd4 is necessary for correct accumulation of differentiated OLs, timely onset of myelination, and accurate myelin thickness. These results agree with and expand findings of studies carried out in parallel by others (Cristobal et al., 2022; Ding et al., 2020). In the SC lineage, we discovered that Nedd4 is required for timely radial sorting of axons and concomitantly for the timely onset of myelination. Moreover, Nedd4 ensures correct myelin thickness.

## 2 MATERIALS AND METHODS

### 2.1 Experimental animals

To achieve conditional deletion of Nedd4 in OLs, Nedd4 floxed mice (*Nedd4* ^*tm3.1Bros*^, MGI:4836405, abbreviated as Nedd4^fl/fl^ in this manuscript) (Kawabe et al., 2010) were crossed with mice carrying a cre recombinase (Cre) transgene under the control of the *Olig2* promoter (*Olig2* ^*tm2(TVA,cre)Rth*^, RRID:IMSR_JAX:011103, abbreviated as Olig2^cre^ in this manuscript; knock‐in allele in the endogenous locus) (Schüller et al., 2008) or *Cnp* promoter (*Cnp* ^*tm1(cre)KAN*^, MGI:3051635, abbreviated as Cnp^cre^ in this manuscript; knock‐in allele in the endogenous locus) (Genoud et al., 2002; Lappe‐Siefke et al., 2003). These breedings gave rise to Olig2^cre^:Nedd4^fl/fl^ (referred to as *Olig2*Nedd4^cKO^) and to Nedd4^fl/fl^ (referred to as Control) mice, or to Cnp^cre^:Nedd4^fl/fl^ (referred to as *Cnp*Nedd4^cKO^) and to Nedd4^fl/fl^ (referred to as Control) mice. The *Gt(ROSA)26Sor* ^*tm1(EYFP)Cos*^ reporter mouse line (RRID:IMSR_JAX:006148, abbreviated as Rosa26‐stop^fl/wt^‐YFP in this manuscript) (Srinivas et al., 2001) was crossed in for fluorescence‐activated cell sorting (FACS)‐based experiments to label recombined cells. In such experiments, Olig2^cre^:Rosa26‐stop^fl/wt^‐YFP mice were used as controls (referred to as Control*) and Olig2^cre^:Nedd4^fl/fl^:Rosa26‐stop^fl/wt^‐YFP mice as mutants (referred to as *Olig2*Nedd4^cKO^*). To achieve conditional deletion of Nedd4 in SCs, Nedd4^fl/fl^ mice were crossed with mice carrying a Cre transgene under the control of *Mpz* (Tg(Mpz‐cre)26Mes/J; RRID:IMSR_JAX:017927, abbreviated as Mpz^cre^ in this manuscript) (Feltri et al., 1999) or *Dhh* (FVB(Cg)‐Tg(Dhh‐cre)1Mejr/J; RRID:IMSR_JAX:012929, abbreviated as Dhh^cre^ in this manuscript) (Jaegle et al., 2003) gene regulatory sequences. These breedings gave rise to Mpz^cre^:Nedd4^fl/fl^ (referred to as *Mpz*Nedd4^cKO^) and to Nedd4^fl/fl^ (referred to as Control) mice, or to Dhh^cre^:Nedd4^fl/fl^ (referred to as *Dhh*Nedd4^cKO^) and to Nedd4^fl/fl^ (referred to as Control) mice.

Genotypes were determined through genomic PCR with the following primers: Nedd4 forward, 5′‐GTA CAT TTT AGT TCA TGG TTC TCA CAG G‐3′; Nedd4 reverse, 5′‐CAG AGG TCA CAT GGC TGT GGG‐3′; Cre forward, 5′‐ATC GCC AGG CGT TTT CTG AGC ATA C‐3′; Cre reverse, 5′ GCC AGA TTA CGT ATA TCC TGG CAG C‐3′; Rosa26‐stop^fl/wt^‐YFP primer A, 5′‐AAA GTC GCT CTG AGT TGT TAT‐3′; Rosa26‐stop^fl/wt^‐YFP primer B, 5′‐GCG AAG AGT TTG TCC TCA ACC‐3′; Rosa26‐stop^fl/wt^‐YFP primer C, 5′‐GGA GCG GGA GAA ATG GAT ATG‐3′. The mouse lines used in this study were on a C57BL/6 background. Cre was used and maintained heterozygous in mice that carry this transgene. Mice were group‐housed with a maximum of five animals/cage, kept in a 12‐h light–dark cycle, and fed standard chow ad libitum. Mice of either sex were used in experiments. Mice were allocated to experiments based on their age and genotype. All animal experiments were performed in accordance with protocols approved by the Zurich Cantonal Veterinary Office (permits ZH090/2017 and ZH119/2020).

### 2.2 Electron microscopy

For spinal cord extraction, mice were euthanized and immediately perfused with 4% paraformaldehyde (Electron Microscopy Sciences Cat. #19208) in 0.1 M phosphate buffer. Spinal cords were dissected and fixed at least overnight with 3% glutaraldehyde (Merck Cat. #G5882) and 4% paraformaldehyde in 0.1 M phosphate buffer. Sciatic nerves were dissected and placed on a thick paper and directly fixed at least overnight with 4% paraformaldehyde and 3% glutaraldehyde in 0.1 M phosphate buffer. After fixation, tissues were treated with 2% osmium tetroxide solution (in 0.1 M phosphate buffer) (Electron Microscopy Sciences, Cat. #19100) and dehydrated over a series of acetone gradients (30%, 50%, 70%, 90%, 96%, and twice at 100%). Following dehydration, acetone was replaced by Spurr's resin (Electron Microscopy Sciences, Cat. #14300) over sequential treatments with increasing resin/acetone gradients (33%, 50%, 66%, 100%). Ultrathin sections (99 nm) of sciatic nerves or of spinal cords within the range of vertebral levels thoracic 13 (T13) and lumbar 1 (L1) were prepared with either a Reichert Jung Ultracut E or a Leica UC7 ultramicrotome and placed on indium tin oxide (ITO) coverslips (Optics Balzers, Cat. #204439). Contrasting of sections was performed with uranyl acetate and lead citrate, and images were acquired with a Zeiss Merlin SEM attached to an ATLAS module. Image acquisition was controlled via the ATLAS software (ATLAS 4 and 5, Zeiss).

### 2.3 Morphological analysis and g‐ratio measurements

For spinal cords, quantification of the number of myelinated and not‐myelinated axons was performed on random fields with equal dimensions overlaid on the electron microscopy (EM) panoramas of similar anatomical regions that were acquired as described above. For analysis at postnatal day 60 (P60), the sums of myelinated axons and clearly identified not‐myelinated axons within these random fields were used to calculate the proportion of myelinated axons. For sciatic nerves, quantifications of sorted axons (number of myelinated and not‐myelinated axons) were performed on whole cross sections of nerves. To obtain measurements for g‐ratio assessment, the axon diameter was derived from the measured axon area, while the fiber diameter was calculated by adding the axon diameter to twice the average myelin thickness measured at two different and well‐preserved locations of the respective myelin ring. From this, the g‐ratio was calculated by dividing the axon diameter by the fiber [axon + myelin] diameter. At least 100 fibers/animal were analyzed in the random fields evaluated (see figure legends for detailed numbers). All measurements were performed using Photoshop versions CC 2018‐2022 (Adobe, RRID:SCR_014199). Additionally, automated quantification of g‐ratio was performed on whole cross sections of sciatic nerves by applying a modified version of AxonSeg (Zaimi et al., 2016) using MATLAB (RRID:SCR_001622). Main changes included the consideration of at least 180 radial projections to evaluate myelin thickness, out of which the average of the second sextile was used for further calculations. Measurements were computationally and manually filtered, yielding at least 1483 g‐ratio values per sample for sciatic nerves.

### 2.4 Antibodies

The following primary antibodies were used: Rabbit anti‐Nedd4 (Cell Signaling Technology, Cat. #2740, RRID:AB_2149312, 1:1000), rabbit anti‐H3 (Cell Signaling Technology, Cat. #4499, RRID:AB_10544537, 1:2000), mouse anti‐α‐Tubulin (Sigma‐Aldrich Cat. #T5168, RRID:AB_477579, 1:10,000), mouse CC1 antibody (Calbiochem, Cat. #OP80, RRID:AB_2057371, 1:500), mouse anti‐OLIG2 (Millipore, Cat. #MABN50, RRID:AB_10807410, 1:1000; used in combination with the PDGFRα antibody and for quantifications of OLIG2+ cells), rabbit anti‐OLIG2 (Millipore, Cat. #AB9610, RRID:AB_570666, 1:500; used in combination with the CC1 antibody), rabbit anti‐PDGFRα (Cell Signaling Technology, Cat. #3174, RRID:AB_2162345, 1:600), goat anti‐SOX10 (R&D System, Cat. #AF2864, RRID:AB_442208, 1:200), rabbit anti‐Ki‐67 (Abcam, Cat. #ab15580, RRID:AB_443209, 1:200; used on sciatic nerve sections), rabbit anti‐Ki‐67 (Abcam, Cat. #ab16667, RRID:AB_302459, 1:200; used on spinal cord sections in combination with the mouse anti‐OLIG2 antibody), rabbit anti‐cleaved caspase 3 (cC3) (Cell Signaling Technology, Cat. #9664, RRID:AB_2070042, 1:1000; on spinal cord sections used in combination with the mouse anti‐OLIG2 antibody). HRP‐, AP‐, and fluorophore‐conjugated secondary antibodies were purchased from Jackson ImmunoResearch and used 1:500 for immunostainings or 1:5000 for western blots.

### 2.5 Immunostainings

For spinal cord extraction, mice were euthanized and subsequently intracardially perfused with PBS (137 mM NaCl, 2.7 mM KCl, 10 mM Na_2_HPO_4_, 1.8 mM KH_2_PO_4_ in H_2_O) followed by 4% paraformaldehyde, 5% sucrose in PBS. Dissected tissues were post‐fixed in a solution containing 4% paraformaldehyde and 5% sucrose in PBS overnight at 4°C, subsequently washed in PBS, cryoprotected with 30% sucrose in PBS overnight at 4°C, and finally embedded in OCT (Sakura, Cat. #4583). Sciatic nerves were dissected immediately after euthanasia and fixed with 4% paraformaldehyde in PBS for 1 h at room temperature, followed by 1 h in 10% sucrose in PBS, and finally overnight in 20% sucrose in PBS at 4°C, before embedding in OCT. Cryosections were prepared at 10 μm and collected on SuperFrost Plus (Thermo Fisher Scientific, Cat. #1255015) microscopy slides, which were air‐dried and stored at −80°C until further use. For spinal cord immunohistochemistry, slides were thawed at room temperature and rehydrated with PBS for 10 min. For sciatic nerve immunohistochemistry, sections were incubated in PBS for 5 min, and then permeabilized (20 min in 0.5% TX100 in PBS) prior to blocking. Slides with spinal cord or sciatic nerve sections were then immersed in blocking buffer (for spinal cords: 1% Triton X‐100, 10% donkey serum, in PBS; for sciatic nerves: 0.1% Triton X‐100, 10% donkey serum, 1% BSA, in PBS) for 1 h and incubated with primary antibody diluted in blocking buffer overnight at 4°C. The following day, sections were washed three times with PBS and incubated with fluorophore‐conjugated secondary antibody diluted in blocking buffer for 1 h at room temperature, counterstained with DAPI (Life Technologies, Cat. #62247), and mounted with ImmuMount (Fisher Scientific, Epredia™, Cat. #10662815). For cC3 immunostaining on spinal cord sections, slides were immersed in blocking buffer (0.3% Triton X‐100, 5% donkey serum, in PBS) for 1 h and incubated with primary antibody diluted in antibody dilution buffer (1% BSA, 0.3% Triton X‐100, in PBS) overnight at 4°C. The following day, sections were washed three times with PBS and incubated with fluorophore‐conjugated secondary antibody diluted in antibody dilution buffer for 1 h at room temperature, counterstained with DAPI (Life Technologies, Cat. #62247), and mounted with ImmuMount (Fisher Scientific, Epredia™, Cat. #10662815). For cell proliferation analysis on sciatic nerve sections, pregnant mice or mouse pups were injected with 50 μg of EdU (Thermo Fisher Scientific, Cat. #A10044)/g of body weight and euthanized 1 h later. EdU staining was performed using the Click‐iT EdU Alexa488 kit (Thermo Fisher Scientific, Cat. #C10337) as per manufacturer's instructions. Immunostainings were imaged using an epifluorescence microscope (Zeiss Axio Imager.M2) equipped with a monochromatic CCD camera (sCMOS, pco.edge), with a ×20 objective. Zen2 Software (blue edition, RRID:SCR_013672) was used for data acquisition. Monochromatic fluorescence images were false colored after acquisition. Three spinal cord hemisections (within the range of vertebral levels T13 and L1) and three sciatic nerve sections per animal were imaged and analyzed. Total spinal cord area analyzed per animal by fluorescence microscopy was 0.58 ± 0.01 mm^2^ for white matter and 1.12 ± 0.02 mm^2^ for gray matter (mean ± standard error of the mean).

### 2.6 Fluorescence‐activated cell sorting of OPC‐ and OL‐enriched fractions

Mice expressing the YFP reporter (Rosa26‐stop^fl/wt^‐YFP) were euthanized at P10, and spinal cords were exposed at the sacral and cervical levels. A syringe fitted with a cut and smoothened canula and filled with cold PBS was inserted into the sacral spinal cavity, allowing extraction of spinal cords from the cervical cavity by hydraulic pressure. Spinal cords were minced and transferred into a dish filled with enzymatic solution (HBSS (Gibco, Cat. #14175‐095), 20 μM HEPES, 80 U/mL DNaseI (Sigma‐Aldrich, Cat. #D4527), 1 mM MgSO_4_, 20 U/mL Papain (Sigma‐Aldrich, Cat. #P3125), 0.46% glucose, 0.5 mM EGTA, 0.24 mg/mL l‐cysteine) previously incubated at 37°C for 30 min. Tissues were treated with the enzymatic solution for 30 min at 37°C on a shaker. The reaction was blocked with Stop buffer (HBSS [Gibco, Cat. #14175‐095], 20 μM HEPES, 10% Fetal Bovine Serum, 40 U/mL DNaseI [Sigma‐Aldrich, Cat. #D4527], 10 mM MgSO_4_, 0.46% glucose, 5 mM EGTA), the dissociated tissue was triturated with a 1 mL pipette tip, passed through a 100 μm SmartStrainer, before transferring into a falcon tube. The suspension was centrifuged at 4°C (5 min at 380*g*). The cell pellet was carefully resuspended in FACS flow buffer (400 μL; 0.5% BSA, 5 mM EGTA, 1× penicillin/streptomycin, 5 μg/mL Insulin, in PBS) and passed through a 50 μm filter. Cell sorting was performed on a Sony SH800 cell sorter (RRID:SCR_018066), and respective software (version 2.1.5).

### 2.7 Oligodendrocyte precursor cell cultures

Mice expressing the YFP reporter (Rosa26‐stop^fl/wt^‐YFP) were used to generate OPC cultures. OPCs were enriched from P10 spinal cords using FACS as described above. Sorted cells were cultured in SATO growth medium (Emery & Dugas, 2013) supplemented with 20 ng/mL PDGFα (Peprotech, Cat. #100‐13A) and 10 ng/mL CNTF (Peprotech, Cat. #450‐13). Cell culture dishes were pre‐coated with 0.02 mg/mL Poly‐d‐lysine (Sigma, Cat. #P6407). Half of the medium was exchanged every other day with full supplement of growth factors. Once confluent, cells were collected for western blot experiments (see below).

### 2.8 RNA extraction for RNA sequencing and RT‐qPCR analysis

Total RNA from acutely sorted OPC‐ and OL‐enriched fractions was extracted using Qiazol (Qiagen, Cat. #79306) as per manufacturer's instructions. Sciatic nerves were placed in ice‐cold PBS, the epineurium and perineurium were removed as much as possible, and nerves were snap‐frozen in liquid nitrogen. Prior to RNA purification, the endoneurium‐enriched nerves were mechanically ground to a powder while frozen using an RNAse‐free pestle (VWR international, Cat. #4310094). Total RNA was extracted using Qiazol as per manufacturer's instructions. Samples were used for RNA sequencing (see section below) or processed for RT‐qPCR analysis as follows: 100 ng of total RNA were reverse transcribed using Maxima First Strand cDNA Synthesis Kit (Thermo Fisher Scientific, Cat. #K1641) as per manufacturer's instructions. qPCR reactions were performed using FastStart Essential DNA Green Master (Roche, Cat. #06402712001) and Light Cycler 480 II (Roche, Product number D 100 03). The following primers were used in the qPCR reactions: Actinb forward, 5′‐GTC CAC ACC CGC CAC C‐3′; Actinb reverse, 5′‐GGC CTC GTC ACC CAC ATA G‐3′; Pdgfra forward, 5′‐CAC AAT AAC GGG AGG CTG GT‐3′; Pdgfra reverse, 5′‐TAT ACA CAG TCT GGC GTG CG‐3′; Enpp6 forward, 5′‐GGA TGG TTT TCG CTC AGA CTA CA‐3′; Enpp6 reverse, 5′‐GTT GCC GAT CAT CTG GTG GA‐3′; Tcf7l2 forward, 5′‐CCT CCG CAC CCT CCA GAT AT‐3′; Tcf7l2 reverse, 5′‐CCT AGA CAT AGA TGC GTT GAC TGT‐3′; Mbp forward, 5′‐CTC CAT CGG GCG CTT CTT TA‐3′; Mbp reverse, 5′‐TGA GTC CTT GTA CAT GTG GCA C‐3′; Mag forward, 5′‐CTG CCT TCA ACC TGT CTG TG‐3′; Mag reverse, 5′‐CGG GTT GGA TTT TAC CAC AC‐3′; Mog forward 5′‐CGT GCA GAA GTA GAG AAT CTC CAT‐3′; Mog reverse, 5′‐ATC ACT CAA AAG GGG TTT CTT AGC T‐3′; Plp1 forward, 5′‐GCA AGG TTT GTG GCT CCA AC‐3′; Plp1 reverse, 5′‐GCG AAG TTG TAA GTG GCA GC‐3′; Klk6 forward, 5′‐CCT GGC AAG ATC ACC CAG AG‐3′; Klk6 reverse, 5′‐GAG GCG ACC CCC ACA TAC TA‐3′. OL‐lineage markers were selected based on (Marques et al., 2016; Xiao et al., 2016).

### 2.9 Illumina RNA‐sequencing and analysis

All steps were performed at the Functional Genomics Center Zurich following the procedure outlined in Gerber et al. (2019) with minor modifications. The quality of isolated RNA was determined with a Qubit (1.0) Fluorometer (Life Technologies) and a Bioanalyzer 2100 (Agilent). Only samples with a 260/280 nm ratio between 1.8–2.1 and a 28S/18S ratio within 1.5–2 were further processed. The TruSeq RNA Sample Prep Kit v2 (Illumina) was used in the succeeding steps. Briefly, total RNA samples (150 ng) were polyA enriched and then reverse‐transcribed into double‐stranded cDNA. The cDNA samples were fragmented, end‐repaired and polyadenylated before ligation of TruSeq adapters containing the index for multiplexing. Fragments containing TruSeq adapters on both ends were selectively enriched with PCR. The quality and quantity of the enriched libraries were validated using Qubit (1.0) fluorometer and the Caliper GX LabChip GX (Caliper Life Sciences). The product was a smear with an average fragment size of approximately 260 bp. Libraries were normalized to 10 nM in 10 mM Tris‐Cl, pH 8.5 with 0.1% Tween 20. The final libraries were sequenced on a NovaSeq 6000 (Illumina) in single read 100 nucleotides mode. The raw reads were first cleaned by removing adapter sequences, trimming low quality ends, and filtering reads with low quality (phred quality <20) using Trimmomatic (Version 0.36, RRID:SCR_011848) (Bolger et al., 2014). Sequence pseudo alignment of the resulting high‐quality reads to the Mouse reference genome (build GRCm38.p6) and quantification of gene level expression (gene model definitions based on GENCODE release M23) was carried out using Kallisto (version 0.44.0, RRID:SCR_016582) (Bray et al., 2016). Differential expression was computed using the generalized linear model implemented in the Bioconductor package edgeR (R version: 3.6.1, edgeR version: 3.28.0, RRID:SCR_012802) (Robinson et al., 2010). Genes showing altered expression with adjusted (Benjamini and Hochberg method) *p*‐value <.05 (labeled as false discovery rate, FDR) were considered differentially expressed. These differentially expressed genes were submitted to gene ontology analysis using EnrichR (2021 version, RRID:SCR_001575) (Chen et al., 2013; Kuleshov et al., 2016) and ShinyGo (version 0.76, RRID:SCR_019213) (Ge et al., 2020), and to the canonical pathway analytical tool of the Ingenuity pathway analysis software (IPA, Qiagen, RRID:SCR_008653; version of July 2022).

### 2.10 Western blots

**FIGURE 1:**
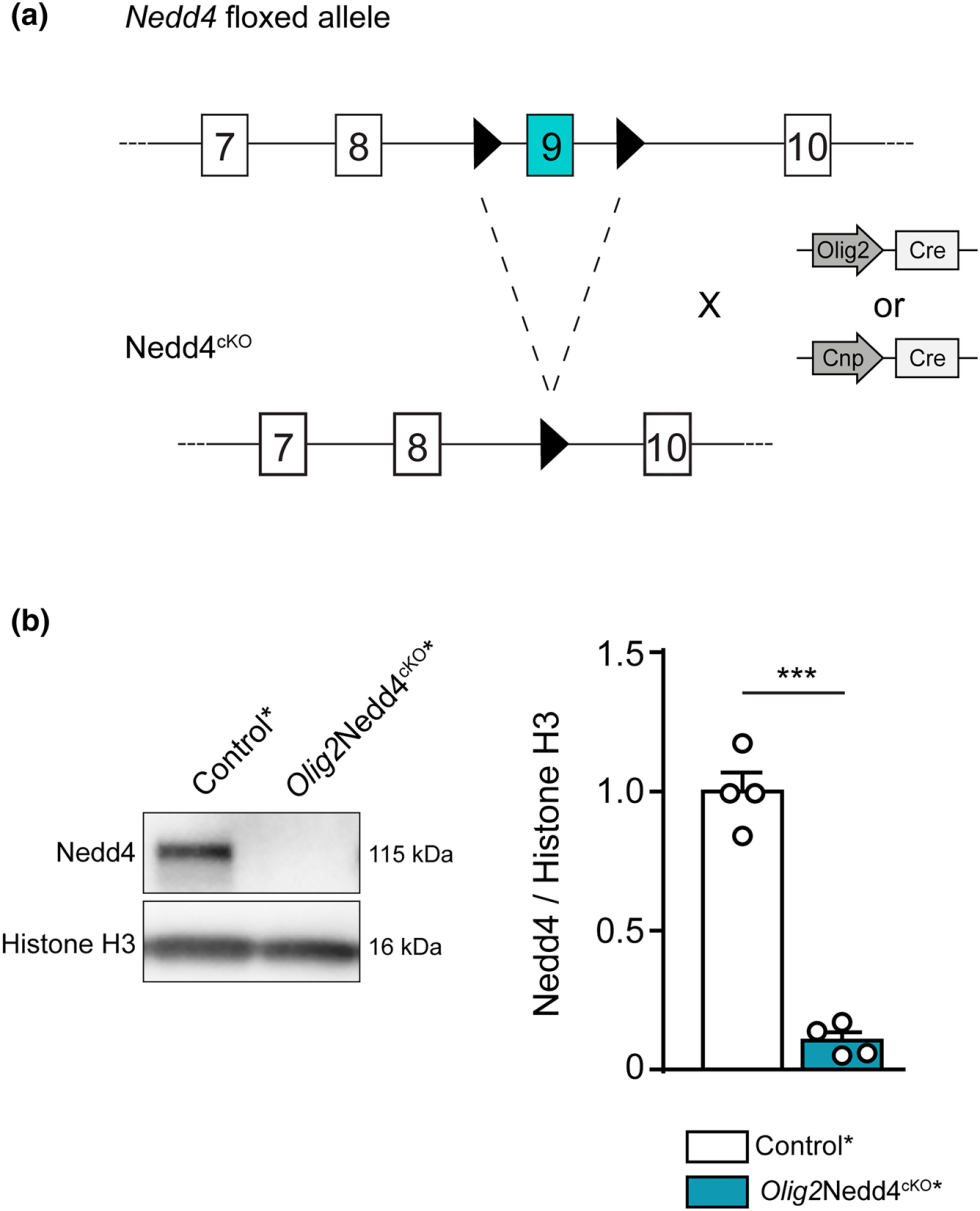
Generation of Nedd4 conditional deletion mouse models in the oligodendrocyte lineage. (a) Schematic of *Nedd4* floxed allele recombination upon *Olig2*‐ or *Cnp*‐driven Cre expression in vivo. *Nedd4* Exon 9 is flanked by two LoxP sites (black arrowheads) (Kawabe et al., 2010). A similar schematic is shown in Figure 6a. (b) Representative picture of western blot analysis of Nedd4 protein levels in primary cultured OPCs enriched by sorting from P10 *Olig2*Nedd4^cKO^* and Control* spinal cords (SpCs), quantified relative to histone H3 level (*n* = independent cultures prepared and analyzed from 4 mice per genotype; ***p < .001; two‐tailed unpaired Student's *t*‐test). Full‐length blots are shown in Figure S2A. Error bars indicate standard error of the mean (SEM). cKO, conditional knock‐out.

To prepare protein lysates from cultured OPCs, cells were harvested in PN2 lysis buffer (25 mM Tris–HCl pH 7.4, 95 mM NaCl, 10 mM EDTA, 2% SDS, 1% protease inhibitor (Roche, Cat. #11844600) and phosphatase inhibitors (Roche, Cat. #04906837001)), sonicated, boiled for 5 min, and centrifuged at 17,000*g* for 10 min at room temperature. To prepare protein lysates from sciatic nerves, these were placed in ice‐cold PBS immediately after dissection, the epineurium and perineurium were removed as much as possible, and finally the endoneurium‐enriched nerves were snap‐frozen in liquid nitrogen and stored at −80°C until further use. To extract proteins, the nerves were ground while frozen as described above, mixed with PN2 lysis buffer, sonicated, boiled for 5 min, and centrifuged at 17,000*g* for 10 min at room temperature. Protein yield was assessed with the Pierce micro‐BCA protein assay kit (Thermo Fisher Scientific, Cat. #23235) following the manufacturer's instructions. Ten μg of proteins/sample were mixed 4:1 with sample buffer (200 mM Tris–HCl pH 6.8, 40% glycerol, 8% SDS, 20% β‐mercaptoethanol, 0.4% bromophenol blue), boiled for 5 min, loaded on 4%–15% polyacrylamide gradient gels (Biorad, Mini Protean TGX) for SDS‐PAGE, and blotted onto a PVDF membrane (Millipore, Cat. #IPVH00010). After blocking with 5% BSA or 5% milk in TBS with 0.1% Tween 20 (TBS‐T), membranes were incubated overnight with primary antibodies diluted in 5% BSA in TBS‐T. The following day, membranes were incubated with HRP‐ or AP‐conjugated secondary antibodies diluted in 5% milk in TBS‐T, 1 h at room temperature. HRP‐ or AP‐conjugated secondary antibodies were visualized using ECL Prime (Amersham, Cat. #GERPN2232) or CDP‐Star (Merck, Cat. #C0712) according to manufacturer's directions, respectively. Signals were detected using Fusion FX7 (Vilber Lourmat). Quantification of band intensities was performed with ImageJ (version 1.53e, RRID:SCR_003070). Histone H3 or α‐Tubulin were used as normalization control. Full‐length western blots related to the bands depicted in figure 1 and figure 6 are shown in Figure S2. The molecular size shown next to the cropped bands refers to the apparent molecular weight estimated from the Precision Plus Protein Standards (BioRad, Cat. #161‐0373).

### 2.11 Quantifications and statistical analysis

Statistical analyses and plotting of graphics were carried out using GraphPad Prism (version 9.2.0, RRID:SCR_002798). Tabular calculations were performed with Microsoft Excel (RRID:SCR_016137). Data distribution was assumed to be normal, and variance was assumed to be equal, although this was not formally tested due to low *n* numbers. Sample sizes were chosen according to sample sizes generally employed in the research field. The following criteria were considered to select the statistical test: two‐tailed unpaired Student's *t*‐test was used when two conditions or genotypes were compared. When one condition was a constant, then the one‐sample *t*‐test was used. A criterion of *p* < .05 was applied for determination of statistical significance. For the analysis of immunohistochemical and morphological data, the investigator was blind to the genotype. No randomization methods were applied. Image levels were adjusted in the same manner across genotypes to improve visibility of the features. Figure assembly was performed in Illustrator CC version 2022 (Adobe, RRID:SCR_010279). Summary of the statistical analysis is available in Table S2.

## 3 RESULTS

### 3.1 Targeting Nedd4 for deletion in the oligodendrocyte lineage

To elucidate the function of Nedd4 in OL development, we carried out loss‐of‐function experiments by breeding mice carrying loxP‐flanked *Nedd4* alleles (Nedd4^fl/fl^) (Kawabe et al., 2010) with mice expressing cre recombinase (Cre) under the control of either *Olig2* (Olig2^cre^) or *Cnp* (Cnp^cre^) gene regulatory elements (Genoud et al., 2002; Lappe‐Siefke et al., 2003; Schüller et al., 2008) (Figure 1a). Within the OL lineage, Olig2^cre^ has been used to achieve recombined floxed sites in OPCs and OLs (Calabretta et al., 2018; Maire et al., 2014; Schüller et al., 2008). Cnp^cre^ leads to recombined floxed sites in some OPCs and in OLs (Goebbels & Nave, 2019; Tognatta et al., 2017). Upon visual inspection, conditional knock‐out mice (*Olig2*Nedd4^cKO^ and *Cnp*Nedd4^cKO^) showed no evident macroscopic differences compared with Controls. Nedd4 protein levels were monitored in OPC‐enriched cell cultures, previously FAC‐sorted from spinal cords (SpCs) of *Olig2*Nedd4^cKO^* or Control* mice at postnatal day (P) 10 (DeGeer et al., 2022) using an additionally bred‐in YFP conditional reporter allele and gating for YFP reporter‐positive OL‐lineage cells and forward scatter area (FSC‐A) (Figure S1A–E). In this setting, YFP reporter expression was used to select for recombined cells, and FSC‐A was used to discriminate OPCs from differentiated OLs by their size. Immunoblotting analysis of protein lysates showed strongly decreased Nedd4 levels in *Olig2*Nedd4^cKO^* compared with Controls* (Figure 1b).

### 3.2 Nedd4 is required for timely onset of myelination and ensures correct myelin thickness in the developing spinal cord of *Olig2*Nedd4^cKO^ mice

**FIGURE 2:**
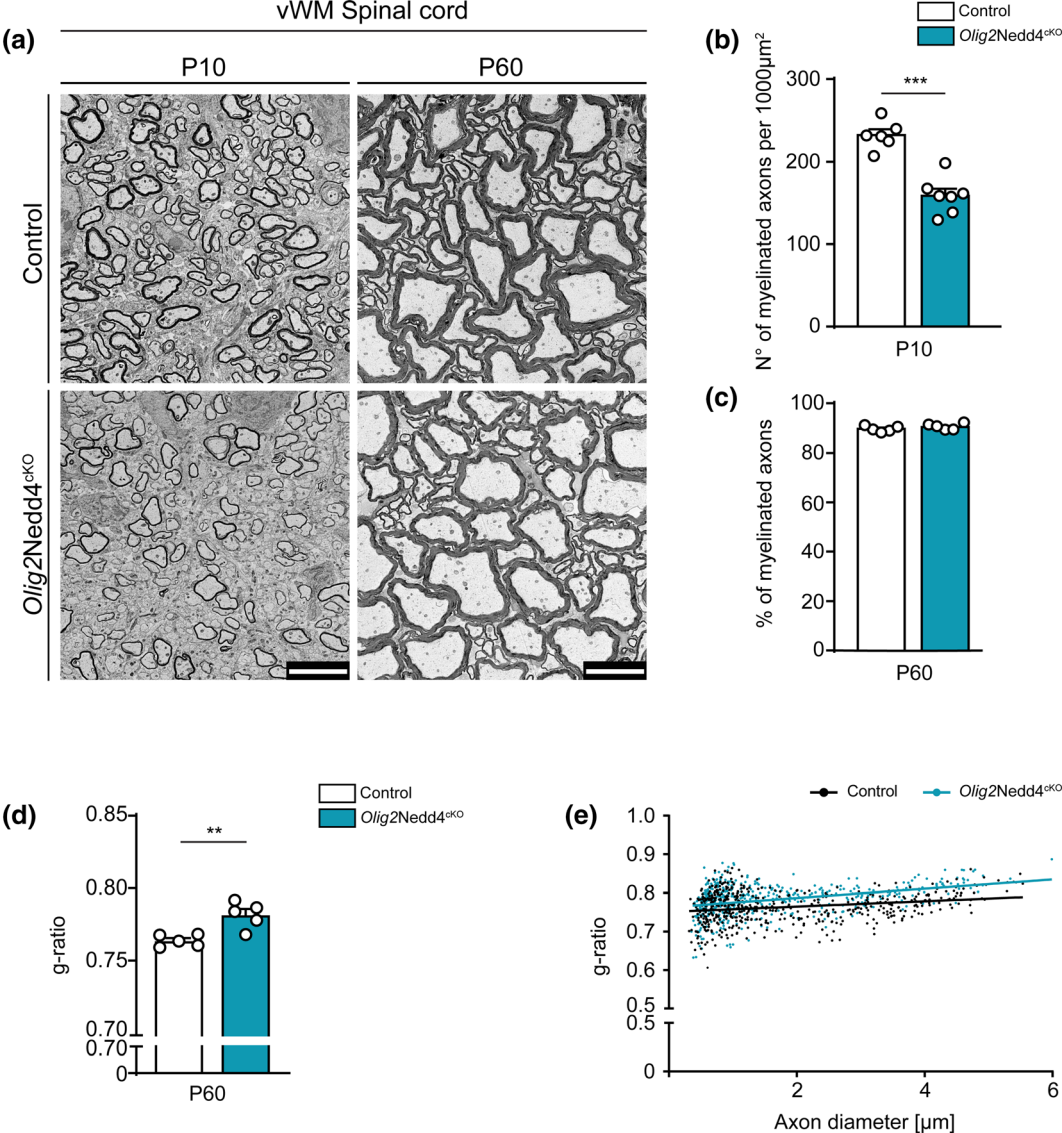
Nedd4 deletion, driven by Olig2^cre^, causes delayed myelination onset and mild hypomyelination in the spinal cord. (a) Representative electron micrographs of Control and *Olig2*Nedd4^cKO^ spinal cord (SpC) ventral white matter (vWM) cross‐sections at different postnatal (P) timepoints. Scale bars: 5 μm. (b) Quantification of the number of myelinated axons per 1000 μm^2^ of SpC vWM in *Olig2*Nedd4^cKO^ compared with Controls at P10 (*n* = 6 and 7 Control and *Olig2*Nedd4^cKO^ mice, respectively. ***p < .001; two‐tailed unpaired Student's *t*‐test; 349–699 axons quantified per animal). (c) Quantification of percentage of myelinated axons in P60 SpC vWM of *Olig2*Nedd4^cKO^ compared with Controls (*n* = 5 mice per genotype; two‐tailed unpaired Student's *t*‐test; 241–545 axons quantified per animal). (d) Quantification of mean g‐ratios of myelinated axons in P60 *Olig2*Nedd4^cKO^ SpC vWM compared with Controls (*n* = 5 mice per genotype; 102–107 axons quantified per animal; **p < .01; two‐tailed unpaired Student's *t*‐test). (e) Scatter plot of measurements and respective linear regression line per genotype showing the distribution of g‐ratio versus axon diameter in SpC vWM of *Olig2*Nedd4^cKO^ compared with Controls at P60 (*n* = 5 mice per genotype). Error bars indicate standard error of the mean (SEM).

To evaluate the impact of Nedd4 on CNS myelination, we carried out morphological analysis of the SpC ventral white matter (vWM) from *Olig2*Nedd4^cKO^ and Control mice at different time points by EM (Figure 2). At P10, we observed fewer myelinated axons per cross‐section area in *Olig2*Nedd4^cKO^ mutants compared with Controls (Figure 2a,b), pointing to a defect in developmental myelination. Further analysis at P60 did not show significant differences in the percentage of myelinated axons in *Olig2*Nedd4^cKO^ mice compared with Controls (Figure 2a,c) indicating that the observed defects at P10 represent a transient delay. To inspect whether Nedd4 deletion affected correct myelin thickness, we quantified the g‐ratio (ratio of axon diameter to fiber [axon + myelin] diameter) at P60. Myelinated axon profiles in *Olig2*Nedd4^cKO^ SpCs displayed a mild but significant increase in g‐ratio values, consistent with a reduction of myelin sheath thickness compared with Controls (Figure 2d,e). Overall, these data indicate that Nedd4 is required for proper developmental myelination and contributes towards ensuring accurate myelin thickness in the developing SpC of *Olig2*Nedd4^cKO^ mice.

### 3.3 Deletion of Nedd4 driven by Olig2^cre^ impedes the correct accumulation of differentiated oligodendrocytes

**FIGURE 3:**
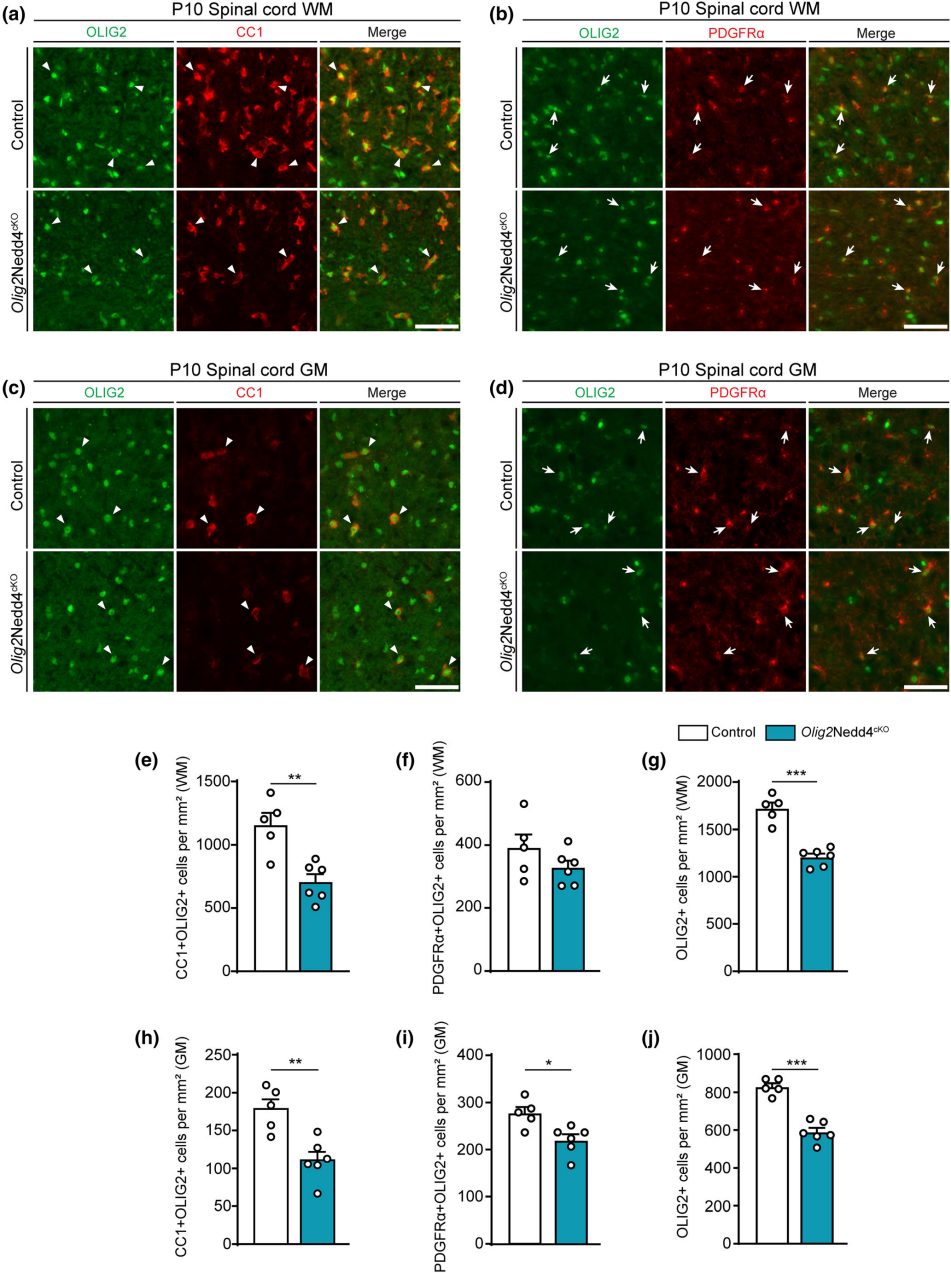
Impaired accumulation of differentiated oligodendrocytes detected after loss of Nedd4, driven by Olig2^cre^, in the spinal cord. (a) Representative immunostainings of P10 *Olig2*Nedd4^cKO^ and Controls in SpC WM cross‐sections labeled with OLIG2 and CC1 antibodies. Arrowheads indicate examples of CC1+ OLIG2+ cells. Scale bar: 50 μm. (b) Representative immunostainings of P10 *Olig2*Nedd4^cKO^ and Controls in SpC WM cross‐sections labeled with OLIG2 and PDGFRα antibodies. Arrows indicate examples of PDGFRα+ OLIG2+ cells. Scale bar: 50 μm. (c) Representative immunostainings of P10 *Olig2*Nedd4^cKO^ and Controls in SpC GM cross‐sections labeled with OLIG2 and CC1 antibodies. Arrowheads indicate examples of CC1+ OLIG2+ cells. Scale bar: 50 μm. (d) Representative immunostainings of P10 *Olig2*Nedd4^cKO^ and Controls in SpC GM cross‐sections labeled with OLIG2 and PDGFRα antibodies. Arrows indicate examples of PDGFRα+ OLIG2 + cells. Scale bar: 50 μm. (e) Quantification of CC1+ OLIG2+ cells per mm^2^ as shown in (a) (**p < .01; two‐tailed unpaired Student's *t*‐test). (f) Quantification of PDGFRα+ OLIG2+ cells per mm^2^ as shown in (b) (two‐tailed unpaired Student's *t*‐test). (g) Quantification of OLIG2+ cells per mm^2^ as shown in (b) (***p < .001; two‐tailed unpaired Student's *t*‐test). (h) Quantification of CC1+ OLIG2+ cells per mm^2^ as shown in (c) (**p < .01; two‐tailed unpaired Student's *t*‐test). (i) Quantification of PDGFRα+ OLIG2+ cells per mm^2^ as shown in (d) (*p < .05; two‐tailed unpaired Student's *t*‐test). (j) Quantification of OLIG2+ cells per mm^2^ as shown in (d) (***p < .001; two‐tailed unpaired Student's *t*‐test). (a–j) *n* = 5 and 6 Control and *Olig2*Nedd4^cKO^ mice, respectively. Error bars indicate standard error of the mean (SEM).

A study involving transgenic mice in different experimental settings has recently reported a role of Nedd4 in OL‐lineage development (Ding et al., 2020). In this context, we evaluated whether Nedd4 is required for the correct accumulation of differentiated OLs in *Olig2*Nedd4^cKO^ mice by carrying out immunohistochemistry on *Olig2*Nedd4^cKO^ and Control SpC cross‐sections at P10 (Figure 3). Our results show a decreased density of differentiated OLs (CC1+ OLIG2+) both in white matter (WM) and gray matter (GM) of *Olig2*Nedd4^cKO^ SpCs compared with Controls (Figure 3a,c,e,h), whereas the density of OPCs (PDGFRα+ OLIG2+) was not significantly changed in WM (Figure 3b,f) and was moderately reduced in GM (Figure 3d,i). Furthermore, we found that the density of all OL‐lineage cells (OLIG2+) was reduced in WM and GM (Figure 3g,j), in line with the observed reduction in the density of differentiated CC1+ OLIG2+ cells. In addition, we assessed proliferation (density of Ki‐67+ OLIG2+ cells) and apoptosis (density of cC3+ OLIG2+ cells). No significant changes in *Olig2*Nedd4^cKO^ SpC WM and GM were detected compared with Controls (Figure S3A–H). These results are consistent with similar findings reported by Ding et al. in a different OL‐lineage Nedd4 cKO mouse model (Nedd4 deletion mediated by Sox10^Cre^) analyzed at an earlier time point (P0) (Ding et al., 2020). Of note, *Olig2* function has been described to be dosage‐dependent during early OL development in an *Olig2* heterozygous knock‐out mouse line with at least partial compensation later (Liu et al., 2007). Another study analyzing a different *Olig2* heterozygous knock‐out line did not report such a dependency (Wedel et al., 2020). In this context, we assessed whether the presence of only one functional allele of *Olig2* in *Olig2*Nedd4^cKO^ mice is likely to contribute to the findings in our specific experimental settings. A comparative analysis of Olig2^cre^ mice with Control (wildtype) mice did not detect significant changes in the density of OLIG2+ cells in SpC WM and GM at P10 (Figure S4E,F,I,J). Moreover, analysis of differentiated OL (CC1+ OLIG2+) and OPC (PDGFRα+ OLIG2+) densities did not reveal significant differences between Olig2^cre^ and wildtype mice in the SpC WM and GM (Figure S4A–H). Taken together, our results agree with and support published findings (Cristobal et al., 2022; Ding et al., 2020) indicating that one function of Nedd4 is its requirement for correct OL‐lineage development.

### 3.4 Deletion of Nedd4 driven by Cnp^cre^ interferes with timely onset of myelination and affects myelin thickness in the developing spinal cord

**FIGURE 4:**
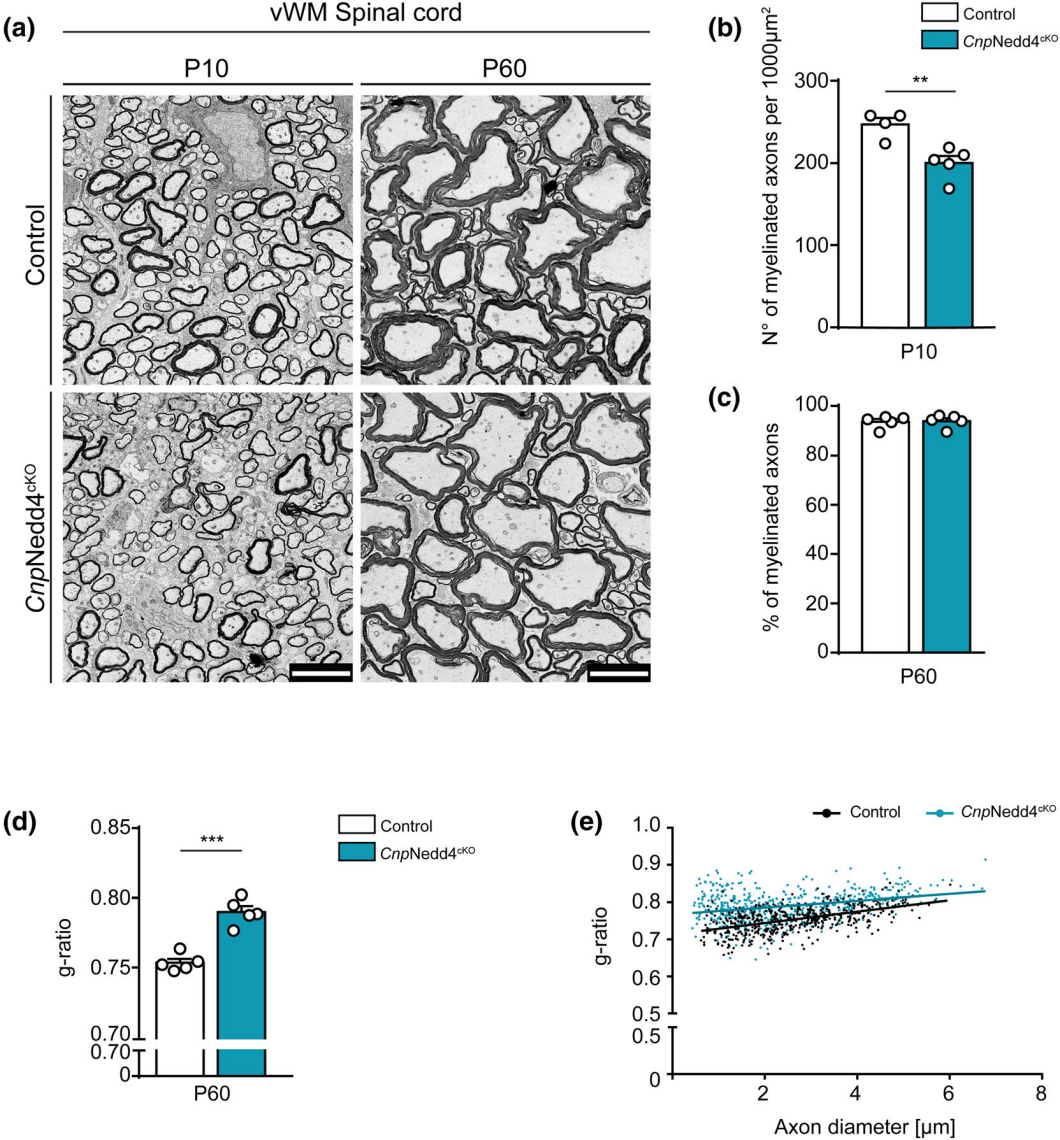
Nedd4 deletion, driven by Cnp^cre^, leads to delayed onset of myelination and mild hypomyelination in the spinal cord. (a) Representative electron micrographs of Control and *Cnp*Nedd4^cKO^ SpC vWM, at different postnatal timepoints. Scale bars: 5 μm. (b) Quantification of number of myelinated axons per 1000 μm^2^ in P10 SpC vWM in *Cnp*Nedd4^cKO^ compared with Controls (*n* = 4 and 5 Control and *Cnp*Nedd4^cKO^ mice, respectively; **p < .01; two‐tailed unpaired Student's *t*‐test; 456–698 axons quantified per animal). (c) Quantification of the percentage of myelinated axons in P60 SpC vWM of *Cnp*Nedd4^cKO^ compared with Controls (*n* = 5 mice per genotype; two‐tailed unpaired Student's *t*‐test; 385–628 axons quantified per animal). (d) Quantification of mean g‐ratio of myelinated axons in P60 *Cnp*Nedd4^cKO^ SpC vWM compared with Controls (*n* = 5 mice per genotype; 101–111 axons quantified per animal; ***p < .001; two‐tailed unpaired Student's *t*‐test). (e) Scatter plot of measurements and respective linear regression line per genotype showing the distribution of g‐ratio versus axon diameter in *Cnp*Nedd4^cKO^ compared with Controls at P60 (*n* = 5 mice per genotype). Error bars indicate standard error of the mean (SEM).

To support the observations in *Olig2*Nedd4^cKO^ mice, we extended our studies to SpCs extracted from *Cnp*Nedd4^cKO^ mice and respective Controls. Comparable to the results in *Olig2*Nedd4^cKO^ mice (Figure 2), we found also: (1) Reduced numbers of myelinated axons per area in *Cnp*Nedd4^cKO^ SpC vWM at P10 (Figure 4a,b); (2) no significant difference in the percentage of myelinated axons between *Cnp*Nedd4^cKO^ SpC vWM and Controls at P60 (Figure 4a,c); and (3) mildly higher g‐ratio values in *Cnp*Nedd4^cKO^ SpC vWM compared with Controls (P60) (Figure 4d,e). Thus, the morphological evaluation of *Cnp*Nedd4^cKO^ mice supports that Nedd4 is required for proper SpC myelination.

**FIGURE 5:**
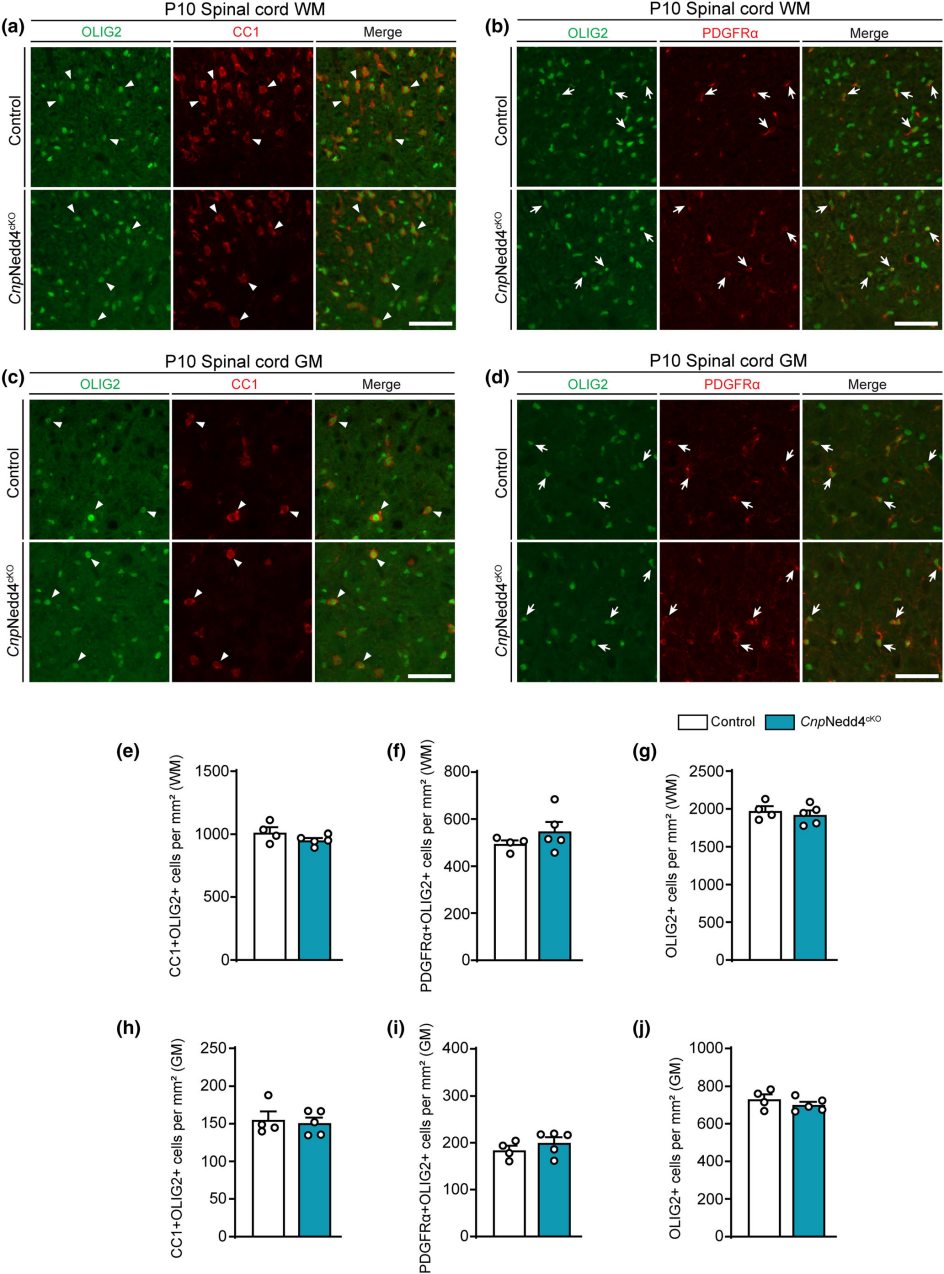
No significant impact on correct accumulation of differentiated oligodendrocytes detected after loss of Nedd4, driven by Cnp^cre^, in the spinal cord. (a) Representative immunostainings of P10 *Cnp*Nedd4^cKO^ and Controls on SpC WM cross‐sections labeled with OLIG2 and CC1 antibodies. Arrowheads indicate exemplary CC1+ OLIG2+ cells. Scale bar: 50 μm. (b) Representative immunostainings of P10 *Cnp*Nedd4^cKO^ and Controls in SpC WM cross‐sections labeled with OLIG2 and PDGFRα antibodies. Arrows indicate exemplary PDGFRα+ OLIG2+ cells. Scale bar: 50 μm. (c) Representative immunostainings of P10 *Cnp*Nedd4^cKO^ and Controls in SpC GM cross‐sections labeled with OLIG2 and CC1 antibodies. Arrowheads indicate exemplary CC1+ OLIG2+ cells. Scale bar: 50 μm. (d) Representative immunostainings of P10 *Cnp*Nedd4^cKO^ and Controls in SpC GM cross‐sections labeled with OLIG2 and PDGFRα antibodies. Arrows indicate exemplary PDGFRα+ OLIG2+ cells. Scale bar: 50 μm. (e) Quantification of CC1+ OLIG2+ cells per mm^2^ as shown in (a) (two‐tailed unpaired Student's *t*‐test). (f) Quantification of PDGFRα+ OLIG2+ cells per mm^2^ as shown in (b) (two‐tailed unpaired Student's *t*‐test). (g) Quantification of OLIG2+ cells per mm^2^ as shown in (b) (two‐tailed unpaired Student's *t*‐test). (h) Quantification of CC1+ OLIG2+ cells per mm^2^ as shown in (c) (two‐tailed unpaired Student's *t*‐test). (i) Quantification of PDGFRα+ OLIG2+ cells per mm^2^ as shown in (d) (two‐tailed unpaired Student's *t*‐test). (j) Quantification of OLIG2+ cells per mm^2^ as shown in (d) (two‐tailed unpaired Student's *t*‐test). (a–j) *n* = 4 and 5 Control and *Cnp*Nedd4^cKO^ mice, respectively. Error bars indicate standard error of the mean (SEM).

We also assessed whether differentiated OLs accumulated correctly in SpCs of *Cnp*Nedd4^cKO^ mice analogously to the immunohistochemical analysis carried out in *Olig2*Nedd4^cKO^ mice (Figure 3). However, we did not find significant differences compared with Controls (Figure 5). Taking also the findings by others on the critical role of Nedd4 in OL‐lineage differentiation into account (Ding et al., 2020), we favor the hypothesis that the lack of a detected deficiency in OL density in *Cnp*Nedd4^cKO^ mice at P10 is likely due to technical aspects, including potential differences in the extent and timing of loss of Nedd4 function in (differentiating) OPCs between our two genetic models. However, the observed comparable effects of loss of Nedd4 on SpC myelination in both *Cnp*Nedd4^cKO^ mice and *Olig2*Nedd4^cKO^ mice, without an overt defect in the density of differentiated OLs in P10 *Cnp*Nedd4^cKO^ mice, are consistent with a potential role of Nedd4 also at later stages of OL development and maturation (Cristobal et al., 2022; Ding et al., 2020).

### 3.5 Targeting Nedd4 for deletion in the Schwann cell lineage

**FIGURE 6:**
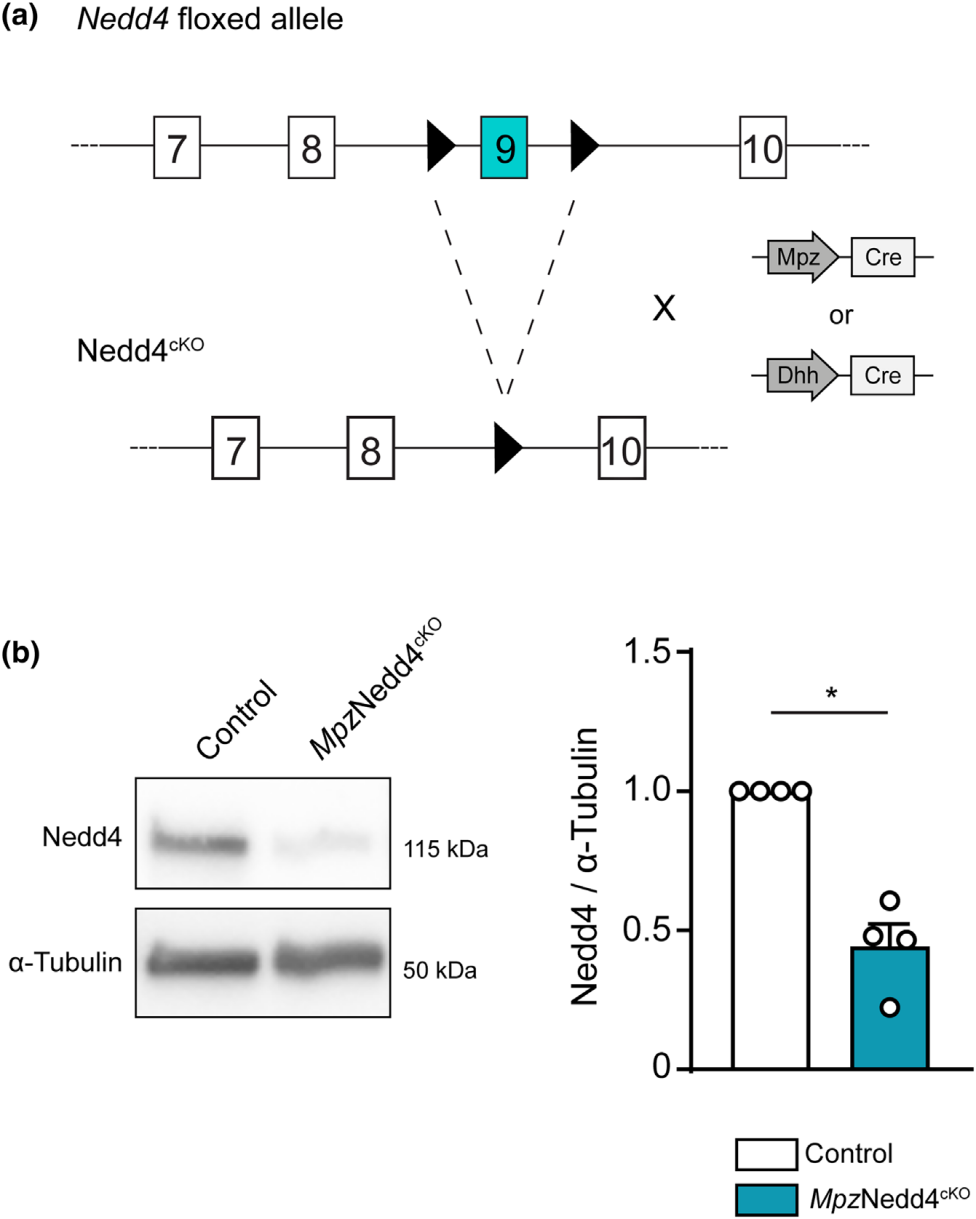
Generation of mouse models with Nedd4 deletion in the Schwann cell lineage. (a) Schematic of *Nedd4* floxed allele recombination upon *Mpz*‐ or *Dhh*‐driven Cre expression in vivo. *Nedd4* Exon 9 is flanked by two LoxP sites (black arrowheads) (Kawabe et al., 2010). A similar schematic is shown in Figure 1a. (b) Representative picture of western blot detection of Nedd4 protein levels in P5 *Mpz*Nedd4^cKO^ sciatic nerves (SNs) compared with Controls, and further quantification of Nedd4 protein levels relative to α‐Tubulin (*n* = 4 mice per genotype; *p < .05; one‐sample *t*‐test). Full‐length blots are shown in Figure S2B. Error bars indicate standard error of the mean (SEM). cKO, conditional knock‐out.

To investigate if Nedd4 has a role in SC development and function, we performed loss‐of‐function experiments by breeding mice carrying loxP‐flanked *Nedd4* alleles (Kawabe et al., 2010) with mice expressing Cre under the control of either *Mpz* (Mpz^cre^) (Feltri et al., 1999) or *Dhh* (Dhh^cre^) (Jaegle et al., 2003) gene regulatory elements (Figure 6a). Reduction of Nedd4 protein levels (Figure 6b) was confirmed in P5 sciatic nerves (SNs) of *Mpz*Nedd4^cKO^ compared with Controls. Note that Nedd4 is also expressed by cell types other than SCs in SNs (Gerber et al., 2021; Yim et al., 2022), a likely explanation for at least some of the residual expression observed in SC‐specific mutants.

### 3.6 Nedd4 in Schwann cells is required for timely radial sorting of axons and ensures correct myelin thickness in the developing sciatic nerve

**FIGURE 7:**
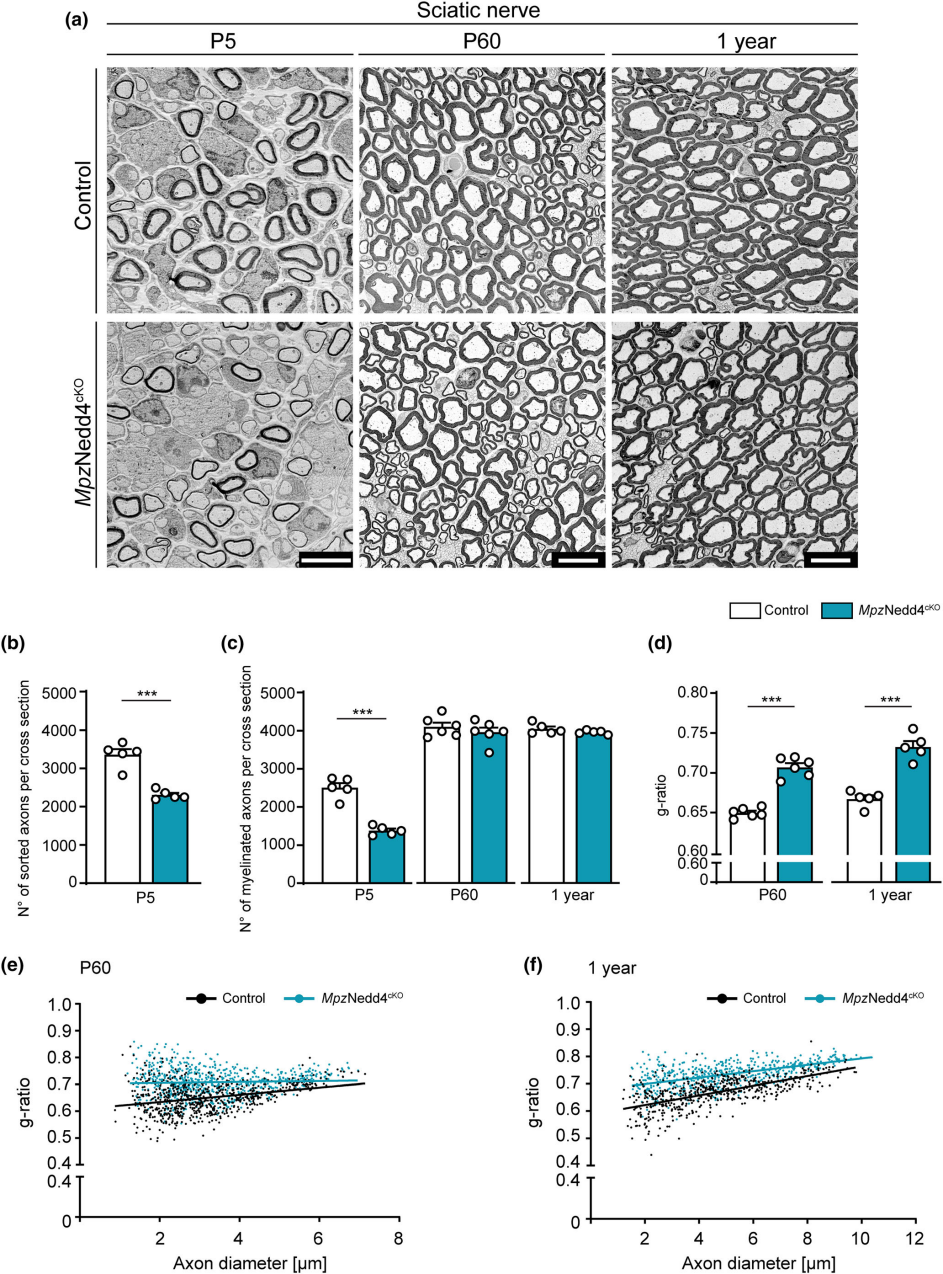
Nedd4 deletion, driven by Mpz^cre^, causes a transient radial sorting impairment and mild myelin thickness defects in the sciatic nerve. (a) Representative electron micrographs of Control and *Mpz*Nedd4^cKO^ SNs at P5, P60 and 1 year. Scale bars: P5, 5 μm; P60 and 1 year, 10 μm. (b) Quantification of number of sorted axons per cross section in P5 SNs from *Mpz*Nedd4^cKO^ compared with Controls (*n* = 5 mice per genotype. ***p < .001; two‐tailed unpaired Student's *t*‐test). (c) Quantification of the numbers of myelinated axons per cross section of P5, P60 and 1 year‐old *Mpz*Nedd4^cKO^ compared with Control SNs (P5 and 1 year: *n* = 5 mice per genotype; P60: *n* = 6 mice per genotype; ***p < .001; two‐tailed unpaired Student's *t*‐test). (d) Quantification of mean g‐ratios of myelinated axons in P60 and 1 year‐old *Mpz*Nedd4^cKO^ SNs compared with Controls (P60: *n* = 6 mice per genotype, 105–128 axons quantified per animal; 1 year: *n* = 5 mice per genotype, 105–111 axons quantified per animal; ***p < .001; two‐tailed unpaired Student's *t*‐test). (e, f) Scatter plot of measurements and respective linear regression line per genotype showing the distribution of g‐ratio versus axon diameter in SNs of *Mpz*Nedd4^cKO^ compared with Controls at P60 (e, *n* = 6 mice per genotype) and 1 year (f, *n* = 5 mice per genotype). Error bars indicate standard error of the mean (SEM).

**FIGURE 8:**
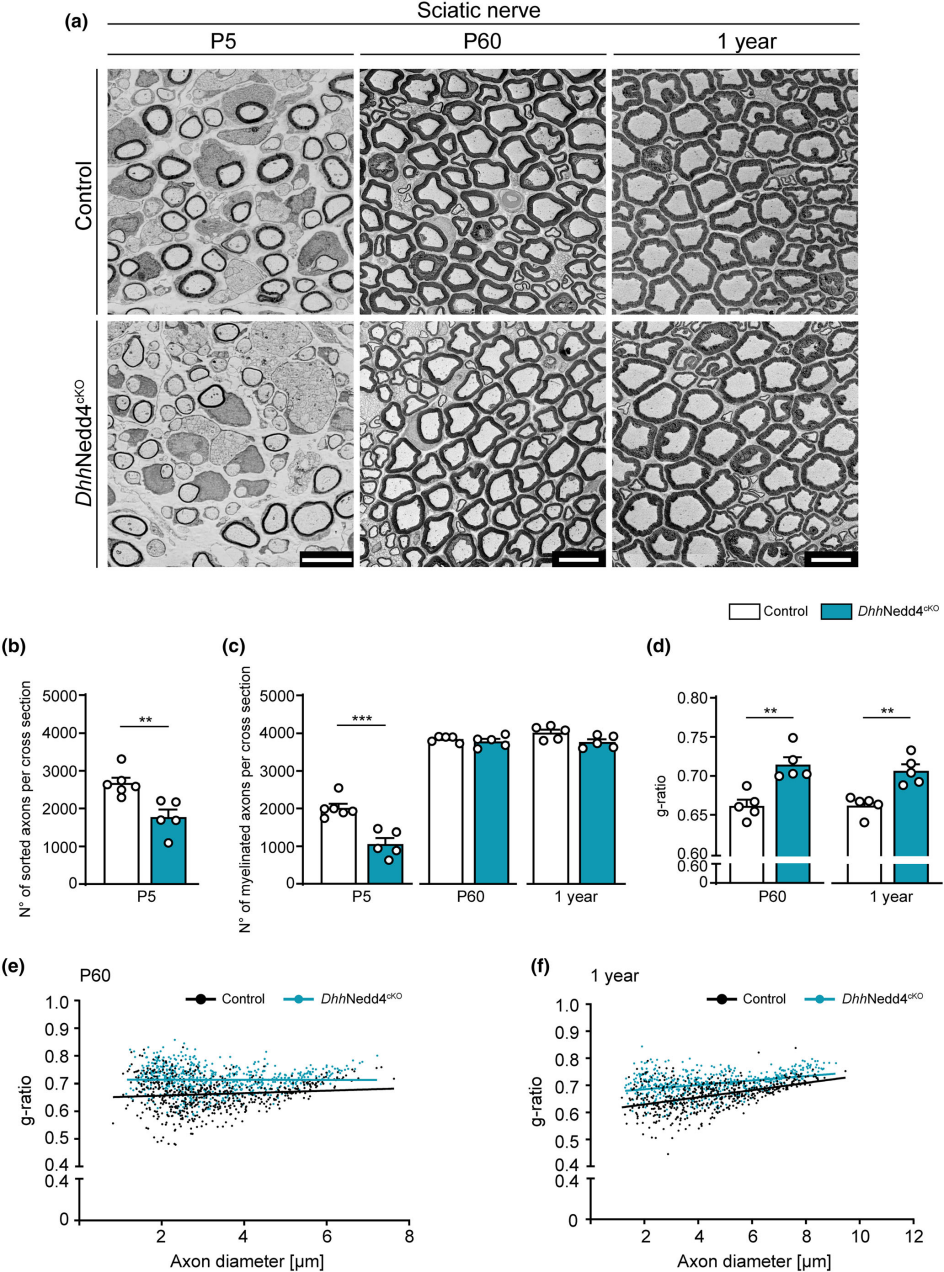
Nedd4 deletion, driven by Dhh^cre^, recapitulates a transient radial axon sorting impairment and mild myelin thickness defects in the sciatic nerve. (a) Representative electron micrographs of Control and *Dhh*Nedd4^cKO^ SNs at P5, P60 and 1 year. Scale bars: P5, 5 μm; P60 and 1 year, 10 μm. (b) Quantification of number of sorted axons per cross section in P5 SNs from *Dhh*Nedd4^cKO^ compared with Controls (*n* = 6 Control and 5 *Dhh*Nedd4^cKO^ mice. **p < .01; two‐tailed unpaired Student's *t*‐test). (c) Quantification of the numbers of myelinated axons per cross section in P5, P60 and 1 year‐old *Dhh*Nedd4^cKO^ SNs compared with Controls (P5: *n* = 6 Control and 5 *Dhh*Nedd4^cKO^ mice; P60 and 1 year: *n* = 5 mice per genotype; ***p < .001; two‐tailed unpaired Student's *t*‐test). (d) Quantification of mean g‐ratios of myelinated axons in P60 and 1 year‐old *Dhh*Nedd4^cKO^ SNs compared with Controls (*n* = 5 mice per genotype, 101–129 axons quantified per animal; **p < .01; two‐tailed unpaired Student's *t*‐test). (e, f) Scatter plot of measurements and respective linear regression line per genotype showing the distribution of g‐ratio versus axon diameter in SNs of *Dhh*Nedd4^cKO^ compared with Controls at P60 (e, *n* = 5 mice per genotype) and 1 year (f, *n* = 5 mice per genotype). Error bars indicate standard error of the mean (SEM).

To evaluate the impact of Nedd4 deletion in SCs in PNS development, we performed morphometric quantifications of SNs from *Mpz*Nedd4^cKO^ or Control mice at different timepoints by EM (Figure 7). At P5, we observed fewer sorted axons (total numbers of single axons surrounded by a SC, myelinated or not) in *Mpz*Nedd4^cKO^ mutants compared with Controls (Figure 7a,b), suggesting a defect in the radial sorting process. Moreover, we found a reduction in the numbers of myelinated axons in *Mpz*Nedd4^cKO^ mutants compared with Controls (Figure 7a,c). Analysis at later timepoints (P60 and 1 year), showed no differences in number of myelinated axons in *Mpz*Nedd4^cKO^ mutants compared with Controls (Figure 7a,c), indicating that the defect observed at P5 has been resolved with time. To assess whether deletion of Nedd4 in SCs affects accurate myelin thickness, we quantified the g‐ratio in SNs from *Mpz*Nedd4^cKO^ and Controls at P60 and 1 year of age. In manual quantifications, *Mpz*Nedd4^cKO^ SNs displayed a mild increase in g‐ratio values at both ages, consistent with a reduction of the myelin sheath thickness compared with Controls (Figure 7a,d–f). As a complementary approach that facilitates analysis of greater numbers of axon‐myelin profiles, g‐ratios were also automatically determined based on myelinated profiles from whole cross sections of SNs using a modified version of AxonSeg (Zaimi et al., 2016). The results agreed with the manual g‐ratio analysis, confirming hypomyelination in P60 and 1 year‐old *Mpz*Nedd4^cKO^ nerves compared with Controls (Figure S5). Note that g‐ratio values obtained by the automated approach are lower than the corresponding manual g‐ratio quantifications, but the differences between genotypes are consistently detectable with both analytic strategies. To gather confirmatory evidence, we extended our analysis to *Dhh*Nedd4^cKO^ mice (Figure 8). Morphological findings in *Dhh*Nedd4^cKO^ SNs were consistent with those in *Mpz*Nedd4^cKO^ animals, revealing reduced numbers of sorted axons and decreased numbers of myelinated axons compared with Controls, defects that disappeared over time (Figure 8a–c). At P60 and 1 year of age, we manually evaluated myelin thickness via g‐ratio analysis. This assessment yielded mildly higher values in *Dhh*Nedd4^cKO^ SNs compared with Controls (Figure 8d–f), indicating mild hypomyelination in *Dhh*Nedd4^cKO^ mutants comparable to the results obtained with *Mpz*Nedd4^cKO^ mice. Taken together, our data from both *Mpz*Nedd4^cKO^ and *Dhh*Nedd4^cKO^ indicate that Nedd4 plays a crucial role in two key aspects of PNS myelination. First, Nedd4 in SCs is required for timely radial sorting of axons and timely onset of myelination. The corresponding defects observed in SC‐Nedd4 cKO mutants were transient and resolved by P60. Second, Nedd4 is needed to ensure correct myelin thickness as indicated by thinner myelin relative to the diameter of associated axon profiles up to 1 year of age in SC‐Nedd4 cKO mutants compared with Controls.

### 3.7 Nedd4 deletion in Schwann cells leads to reduced numbers of developing Schwann cells in the sciatic nerve

**FIGURE 9:**
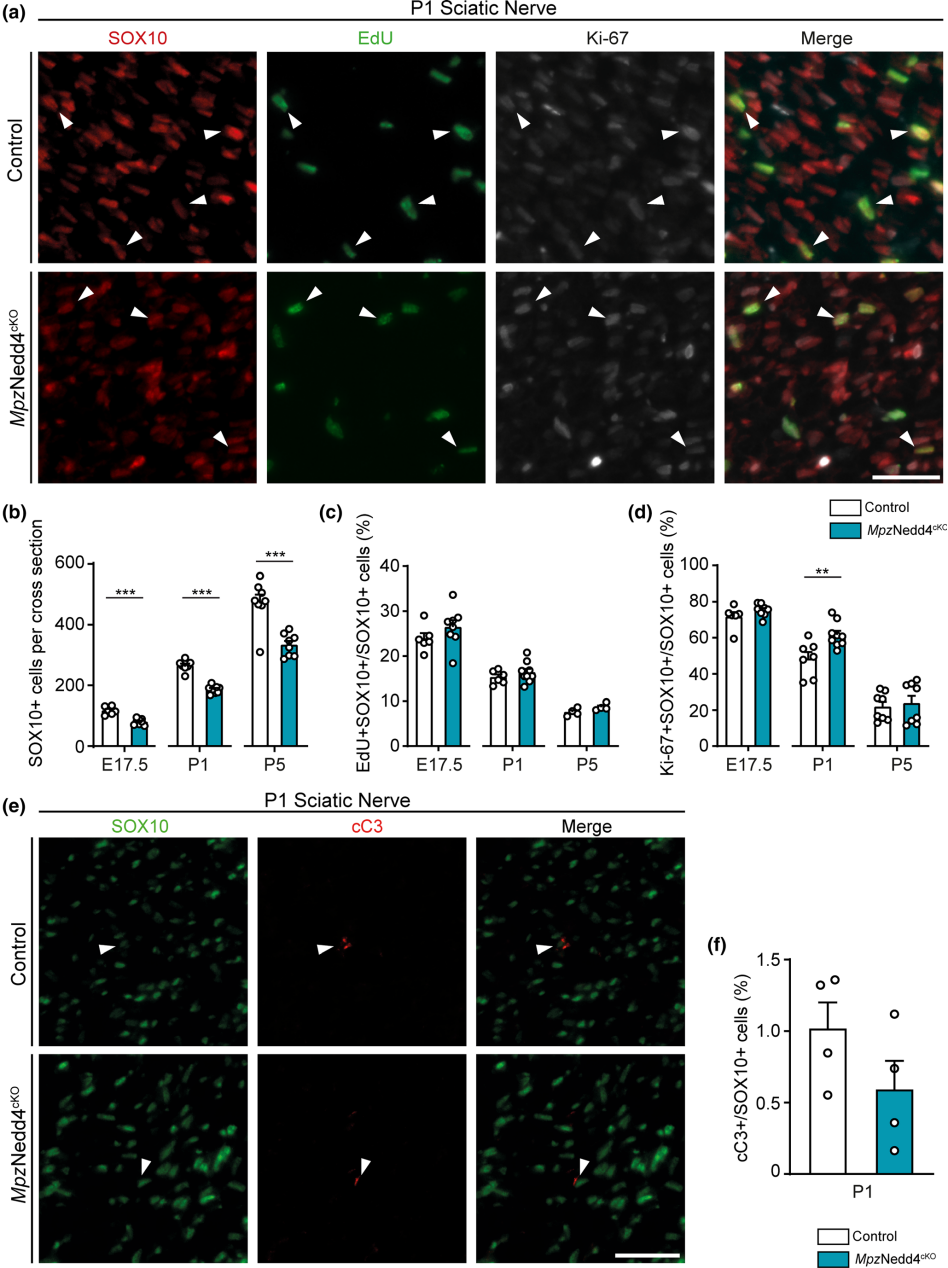
Nedd4 deletion leads to a reduction in Schwann cell numbers in developing *Mpz*Nedd4^cKO^ sciatic nerves. (a) Exemplary immunostainings of P1 *Mpz*Nedd4^cKO^ and Control SN cross‐sections labeled with SOX10 and Ki‐67 antibodies, also showing EdU fluorescent labellings. Arrowheads indicate exemplary EdU+ Ki‐67+ SOX10+ SCs. Scale bar: 50 μm. (b) Quantification of SOX10+ SCs per cross section at P1 as shown in (a), and of additional stainings performed at E17.5 and P5 (including four mice/genotype at P5 not injected with EdU) (E17.5: *n* = 6 Control and 8 *Mpz*Nedd4^cKO^ mice; P1: *n* = 7 Control and 9 *Mpz*Nedd4^cKO^ mice; P5: *n* = 8 mice per genotype. ***p < .001; two‐tailed unpaired Student's *t*‐test). (c) Quantification of the percentage of EdU+ SOX10+/SOX10+ SCs per cross section as shown at P1 in (a), and of additional stainings performed at E17.5 and P5 (E17.5: *n* = 6 Control and 8 *Mpz*Nedd4^cKO^ mice; P1: *n* = 6 Control and 9 *Mpz*Nedd4^cKO^ mice; P5: *n* = 4 mice per genotype. Two‐tailed unpaired Student's *t*‐test). (d) Quantification of percentage of Ki‐67+ SOX10+/SOX10+ SCs per cross section as shown at P1 in (a), and of additional stainings performed at E17.5 and P5 (including four mice/genotype at P5 not injected with EdU) (E17.5: *n* = 6 Control and 8 *Mpz*Nedd4^cKO^ mice; P1: *n* = 7 Control and 9 *Mpz*Nedd4^cKO^ mice; P5: *n* = 8 mice per genotype. **p < .01; two‐tailed unpaired Student's *t*‐test). (e) Representative immunostainings of P1 *Mpz*Nedd4^cKO^ and Controls on SN cross‐sections labeled with SOX10 and cleaved caspase 3 (cC3) antibodies. Arrowheads indicate cC3+ SOX10+ SCs. Scale bar: 50 μm. (f) Quantification of the percentage of cC3+ SOX10+/SOX10+ SCs per cross section as shown in (e) (*n* = 4 mice per genotype; two‐tailed unpaired Student's *t*‐test). Error bars indicate standard error of the mean (SEM).

To ensure correct radial sorting, SCs have to expand sufficiently in numbers to allow the available large caliber axonal segments to become engaged in a 1:1 relationship with them (Jessen & Mirsky, 2005). Reduced SC numbers through defective proliferation or increased cell death may cause defects in radial sorting (Feltri et al., 2016; Previtali, 2021). Thus, we evaluated the numbers of SCs in *Mpz*Nedd4^cKO^ and Control mice using the SC marker SOX10 at different timepoints when radial sorting is ongoing (embryonic day (E) 17.5, P1 and P5) (Figure 9a,b). The obtained data indicate a reduction of SCs in *Mpz*Nedd4^cKO^ compared with Control mice at P5, correlated with our previous morphological observations of impaired radial sorting in the mutants. Moreover, a reduction in SCs was also detectable at earlier ages examined (P1, E17.5; Figure 9b). To determine whether altered proliferation contributed to the changes in SC numbers observed in mutant SNs, we assessed SC proliferation by 5‐ethynyl‐2′‐deoxyuridine (EdU, a label incorporated during S phase) treatment and by Ki‐67 immunostainings (which labels proliferating cells irrespective of the cell cycle phase), together with SOX10 immunostainings (Figure 9a). Throughout the timepoints analyzed (E17.5, P1 and P5), the percentages of both EdU+ or Ki‐67+ SCs (SOX10+) were not significantly reduced in *Mpz*Nedd4^cKO^ compared with Control mice (Figure 9a,c,d). The analysis suggested even a mild increase in the percentage of Ki‐67‐positive SCs (SOX10+) in *Mpz*Nedd4^cKO^ at P1. We next evaluated apoptosis by immunohistochemistry for cleaved caspase 3 (cC3+) in combination with the SC marker SOX10 (Figure 9e,f) in P1 SNs of *Mpz*Nedd4^cKO^ and Control mice, but we found no significant differences in the fractions of cC3+ SCs (Figure 9f). Collectively, these data indicate that *Mpz*Nedd4^cKO^ SNs contain fewer SCs compared with Controls already before and persisting during the main period of axonal sorting, likely contributing to the impairment of this process. However, we could not detect a direct correlation with defects in SC proliferation and/or survival at the timepoints analyzed during axonal sorting. Further studies will be required to determine the reasons for the reduced SC numbers observed in *Mpz*Nedd4^cKO^ SNs and other potential effects of loss of Nedd4 on cell dynamics in SC development.

### 3.8 Transcriptome analysis reveals changes consistent with impaired myelination in *Mpz*Nedd4^cKO^ sciatic nerves

**FIGURE 10:**
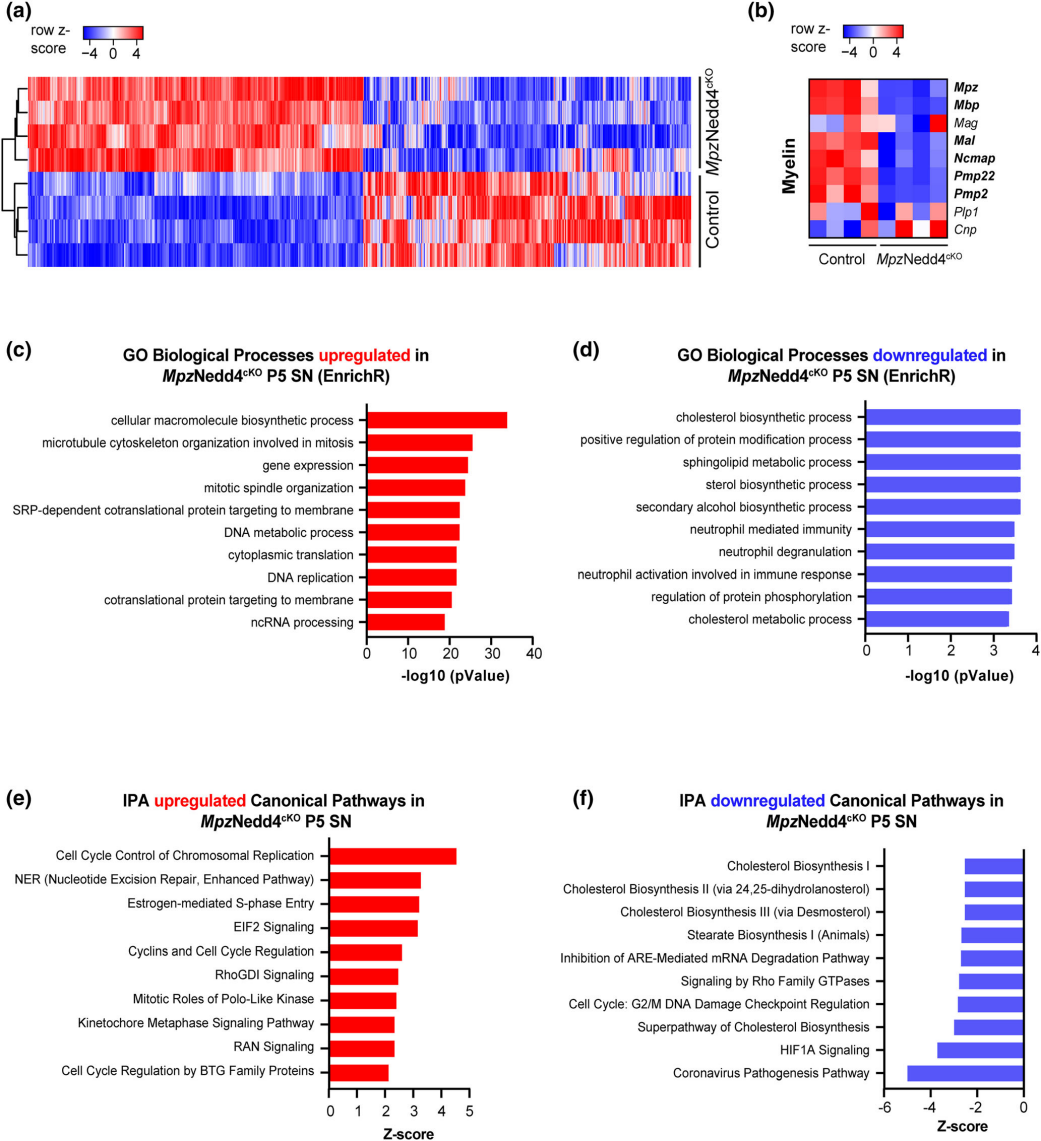
Nedd4 deletion leads to transcriptome changes coherent with delayed developmental myelination in *Mpz*Nedd4^cKO^ sciatic nerves. (a) Heatmap (row *z*‐scores) of significantly differentially expressed genes (FDR <0.05) obtained from *Mpz*Nedd4^cKO^ and Control P5 SNs (*n* = 4 mice per genotype). (b) Heatmap (row *z*‐scores based on normalized transcripts) of selected transcripts encoded by genes related to myelin proteins (*n* = 4 mice per genotype) obtained from *Mpz*Nedd4^cKO^ and Control P5 SNs. In bold are the genes with FDR <0.05. (c) EnrichR Gene ontology (GO) of biological processes (BP) for significantly upregulated transcripts expressed by *Mpz*Nedd4^cKO^ compared with Control P5 SNs (FDR <0.05; *n* = 4 mice per genotype). (d) EnrichR GOBP for significantly downregulated transcripts expressed by *Mpz*Nedd4^cKO^ compared with Control P5 SNs (FDR <0.05; *n* = 4 mice per genotype). (e) Upregulated canonical pathways (ingenuity pathway analysis, IPA) in *Mpz*Nedd4^cKO^ compared with Control P5 SNs (FDR <0.05; *n* = 4 mice per genotype). (f) Downregulated canonical IPA pathways in *Mpz*Nedd4^cKO^ compared with Control P5 SNs (FDR <0.05; *n* = 4 mice per genotype).

Based on the results described above, Nedd4 in SCs plays key roles in promoting radial sorting, likely via establishing correct SC numbers, and in ensuring accurate myelin thickness in SNs. To obtain hints of which molecular pathways are affected upon loss of Nedd4 in SCs, we evaluated changes in the transcriptome by RNA sequencing of *Mpz*Nedd4^cKO^ and Control SNs at P5 (Figure 10). Plotting differentially regulated transcripts revealed clustering of individual samples per genotype (2044 upregulated and 2002 downregulated genes [FDR <0.05]) (Figure 10a). Coherent with the defects in myelination observed in the morphological analysis, we found that genes related to selected myelin proteins (Siems et al., 2020) were expressed at lower levels in *Mpz*Nedd4^cKO^ nerves compared with Controls (Figure 10b). Further analyses using software tools that assess gene ontology (GO) of biological processes (BP) (EnrichR, ShinyGo) and the ingenuity pathway analysis (IPA) consistently revealed cholesterol biosynthesis among the most differentially downregulated categories in *Mpz*Nedd4^cKO^ compared with Control SNs (Figure 10d,f and Figure S6B). These findings are in line with studies that have suggested impaired cholesterol and lipid biosynthesis to be correlated with deficits in PNS myelination (Gerber et al., 2019; Saher et al., 2011; Wüst et al., 2020). Among the most upregulated categories found in *Mpz*Nedd4^cKO^ compared with Controls, terms related to cell cycle and proliferation were consistently present (Figure 10c,e and Figure S6A). Primary data are provided in the GEO database under accession number (GSE217272) and in Table S1 for further data mining.

## 4 DISCUSSION

In this study, we have evaluated multiple mouse models with conditional gene deletions to demonstrate that OL lineage‐ and SC lineage‐expressed E3 ubiquitin ligase Nedd4 is essential for accurate developmental myelination in the CNS and in the PNS.

Our results in the SpC suggest, together and consistent with published complementary findings obtained in different experimental settings including the analysis of OL development using Sox10^Cre^‐ and NG2^CreER^‐mediated Nedd4 deletions (Cristobal et al., 2022; Ding et al., 2020), that Nedd4 may regulate the OL lineage at multiple stages. As shown by Ding et al. (2020), Sox10^Cre^‐mediated deletion of Nedd4 led to a reduction of differentiated OLs in P0 SpCs, whereas Nedd4 overexpression in OPC cell culture increased OL differentiation. Further analysis of the tissue samples revealed no significant changes in proliferation or apoptosis in the OL lineage. These findings are consistent with our observations in *Olig2*Nedd4^cKO^ mice, collectively supporting the interpretation that one function of Nedd4 is to foster correct OL‐lineage differentiation. This does not exclude, however, that Nedd4 may also regulate other cellular processes such as OPC specification and/or proliferation and/or cell death, in particular in early development. Mechanistically, it was previously shown that Nedd4 increases the stability of von Hippel–Lindau (VHL) protein via K63‐linked polyubiquitination (Ding et al., 2020). Like Nedd4, VHL is also a ubiquitin ligase that drives differentiation along the OL lineage, involving ubiquitination and degradation of hypoxia‐inducible factor (HIF) transcription factors (Ding et al., 2020; Yuen et al., 2014). Dishevelled associated activator of morphogenesis 2 (Daam2) antagonizes the pathway and suppresses differentiation of OPCs into OLs, both in development and in remyelination following lysolecithin lesions (Ding et al., 2020). Our results suggest that Nedd4 is required for timely onset of myelination and for ensuring appropriate myelin thickness relative to the associated axons in the developing SpC, consistent with EM findings by Ding et al. (2020) in P14 SpC and corpus callosum after NG2^CreER^‐mediated Nedd4 deletion. Taken together, the available experimental evidence is in line with a potential direct role of Nedd4 in myelination, consistent with our observations that myelination defects occurred not only in developing *Olig2*Nedd4^cKO^ mice which displayed an impairment in the correct accumulation of differentiated OLs, but also in *Cnp*Nedd4^cKO^ mice that did not show evident defects in this context. Furthermore, the later time point (P60) evaluated by EM using both Cre lines in our study shows that the impact of Nedd4 deletion on the onset of myelination is transient and recovers. However, mild hypomyelination was still present at P60 as indicated by increased g‐ratio.

In the PNS, EM analysis of P5 SNs of *Mpz*Nedd4^cKO^ and *Dhh*Nedd4^cKO^ mice revealed that expression of Nedd4 in the SC lineage is required for appropriate radial sorting of axons. During radial sorting, immature SCs select single axonal segments from immature axon bundles to form a 1:1 SC‐axon relationship through a series of steps that involve extension of cytoskeleton‐filled processes into the bundles, cellular recognition, and expansion of the available SCs to match the numbers of axonal segments that need to be sorted (Feltri et al., 2016; Previtali, 2021). To investigate the potential basis of the defects observed in radial sorting in SC‐specific Nedd4‐depleted mice, we focused the analysis at time points known to be particularly relevant for proper radial sorting in SNs of mice (E17.5, P1 and P5) and found fewer SCs in *Mpz*Nedd4^cKO^ SNs already at E17.5. This early deficit likely contributes to the lower numbers of SCs observed also at P1 and P5, in line with our failure of detecting overt major alterations in SC proliferation and cC3‐monitored apoptosis. The impact of Nedd4 deletion has been studied in neural crest cells and found to be associated with a reduction in a subset of these cells, increased apoptosis, and lower levels of SOX10 expression (Lohraseb et al., 2022; Wiszniak et al., 2013). Complementary cell culture experiments pointed to impaired proliferation as another potential contributor (Wiszniak et al., 2013). Given that the SC lineage derives from neural crest cells, it is possible that loss of Nedd4 in SC‐mutant SNs affects SCs at early developmental stages in a related manner, potentially contributing to the fewer SCs observed at the time of radial sorting.

Our results show that SC lineage‐expressed Nedd4 is required to establish correct SC numbers at ages relevant to radial sorting, and we favor the interpretation that reduced SC numbers are a major contributor to the radial sorting defects in SNs containing Nedd4‐depleted SCs. However, it remains possible that loss of Nedd4 might also interfere with other mechanisms involved in axonal sorting such as SC‐axon recognition processes or SC cytoskeleton dynamics (Feltri et al., 2016; Previtali, 2021). Furthermore, in healthy mouse SNs, virtually all progressively sorted 1:1 SC‐axon profiles start myelination. However, we observed fewer myelinated axons in SNs of both *Mpz*Nedd4^cKO^ and *Dhh*Nedd4^cKO^ mice compared with respective Controls at P5. This defect is likely due to the reduced pool of sorted axons and SCs that are available to start myelination, although we cannot rule out that Nedd4 may also have a more direct functional role in the regulation of the onset of SC‐mediated myelination.

In young adults (P60), EM analysis of SNs from *Mpz*Nedd4^cKO^ and *Dhh*Nedd4^cKO^ mice showed no longer significant differences in the number of myelinated axons when compared with respective Controls, indicating that this morphologically observed defect detected in P5 mutant SNs is transient. Additional analyses revealed higher g‐ratio values in myelinated profiles of mutants compared with Controls, indicating that Nedd4 function contributes to ensuring myelin with appropriate thickness relative to the diameter of the associated axonal segments. Moreover, analysis of *Mpz*Nedd4^cKO^ and *Dhh*Nedd4^cKO^ mice at 1 year of age revealed sustained higher g‐ratio values compared with Controls, further supporting essential contributions of Nedd4 towards correct radial myelin proportions. On a technical note, our g‐ratio analyses using manual and automated approaches yielded consistent differences between Control and *Mpz*Nedd4^cKO^ samples, but the values were overall lower with the automated compared with the manual approach. The possibility of such deviations has been noted previously (Moiseev et al., 2019; Saliani et al., 2019). However, the automated approach has the advantage of facilitating the efficient analysis of more myelin‐axon profiles, overall allowing to increase the robustness of the results further.

To monitor transcriptional alterations that might correlate with the morphological observations in *Mpz*Nedd4^cKO^ mice, we performed bulk RNA sequencing using RNA extracted from P5 mutant SNs in comparison with Controls. By submitting the significantly downregulated transcript list to unbiased GOBP analysis, both with EnrichR (Chen et al., 2013; Kuleshov et al., 2016) and ShinyGO (Ge et al., 2020) tools, and to the canonical pathways analysis of IPA, several categories related to cholesterol biosynthesis were consistently identified. We consider it likely that these observations, found in conjunction with significantly reduced levels of transcripts encoding myelin proteins, reflect the delayed developmental myelination status of SNs of *Mpz*Nedd4^cKO^ mice. Additional unbiased analysis of significantly upregulated transcripts yielded several categories related to mitosis and cell cycle regulation in mutants. These findings are of interest and reminiscent of related cell dynamic aspects discussed earlier. However, we have not been successful to identify convincing patterns of consistent specific cellular pathway alterations due to SC‐Nedd4 depletion in this context, possibly also related to the technical approach used (Gerber et al., 2021).

Given its role as E3 ubiquitin ligase, Nedd4 is likely capable of conjugating ubiquitin to a plethora of targets in the healthy SC‐ and OL‐lineages. To further explore which proteins are differentially ubiquitinated if Nedd4 is conditionally deleted, it would be particularly interesting to carry out proteomics analysis of ubiquitinated proteins (Fulzele & Bennett, 2018) as recently done using a neural crest cell line (Lohraseb et al., 2022). Ideally, such a strategy requires extraction of correct cell types from the relevant physiological context. This would entail using Nedd4 conditional knock‐out and Control SCs acutely extracted from SNs undergoing radial sorting, and OL‐lineage cells extracted from SpC undergoing OL differentiation. Our pilot efforts showed that major technical improvements will be required to enable robust experiments, including substantially increased assay sensitivity to reduce the amount of protein needed (Kim et al., 2011; Vere et al., 2020).

In addition to limitations of our study discussed already, we would like to mention some specifics related to the transgenic tools used in the CNS analysis. The Cnp^cre^ and Olig2^cre^ mice that we employed to conditionally disrupt the *Nedd4* gene are well established to achieve recombination involving LoxP sites in the OL lineage (for Cnp^cre^ see MGI:3051635, and for Olig2^cre^ see MGI:3810299) (Goebbels & Nave, 2019). Notably, these Cre lines carry both knock‐in alleles and thus, the respective mutant animals harbor only one functional copy of *Cnp* or *Olig2*. Based on previous studies using these mouse lines, combined with our findings and those of Ding et al. (2020), we consider it unlikely that our restricted interpretations about Nedd4 function are significantly disturbed by these particular genetic settings.

Cnp^cre^ can cause recombined floxed sites in some neurons (Genoud et al., 2002; Goebbels & Nave, 2019; Jo et al., 2021; Tognatta et al., 2017). Concerning the Olig2^cre^ line, indications for recombined floxed sites have been found in some astrocytes and a subset of neurons (Carson et al., 2015; Ju et al., 2016; Schüller et al., 2008; Short et al., 2019; Tsai et al., 2012). We have not examined potential recombination outside of the OL lineage in our settings and cannot formally exclude an influence of such events. However, we have no indication that our interpretations of the results concerning Nedd4 function in OL‐lineage development could be majorly affected. In particular, we have not observed evident signs of axonal/neuronal pathology.

Taken together, despite some limitations and the restricted analyses performed, when considering our findings in conjunction with the study by Ding et al. (2020), four independent Cre lines used to examine the effects of Nedd4 loss on OL‐lineage development have yielded consistent results indicating crucial functions of this ubiquitin ligase in OL biology.

## AUTHOR CONTRIBUTIONS

*Conceptualization*: C.F., J.A.P., and U.S. *Methodology*: C.F., J.A.P., J.G., S.B., J.D., and U.S. *Validation*: C.F., J.G., and J.A.P. *Formal analysis*: C.F., J.G., I.B., J.F., S.B., and M.K. *Investigation*: C.F., J.G., I.B., J.F., M.K., S.B., and J.A.P. *Data curation*: C.F., J.G., and J.A.P. *Writing—original draft*: C.F. and J.A.P. *Writing—review and editing*: C.F., J.A.P., J.G., I.B., J.D., S.B., J.F., M.K., and U.S. *Visualization*: C.F. and J.A.P. *Supervision*: C.F., J.A.P., and U.S. *Resources*: U.S. *Project administration*: U.S.

## Supporting information


**Supplementary Figures**:

**Table S1: Processed RNA sequencing data from postnatal day 5 (P5) sciatic nerves**.
Processed bulk RNA sequencing data of detected transcripts derived from *Mpz*Nedd4^cKO^ and Control sciatic nerves at P5.

**Table S2: Statistical analysis summary**.
Summary of the statistical analysis reported in this study.

## Data Availability

The data that support the findings of this study are available from the corresponding author upon reasonable request. RNA sequencing data are available at the Gene Expression Omnibus (GEO) accession number GSE217272.
