# Supplementary material for: The E3 ubiquitin ligase Nedd4 fosters developmental myelination in the mouse central and peripheral nervous system
Source: Glia. 2024 Nov 7;73(2):422–44. doi: 10.1002/glia.24642 (PMC11662984; doi:10.1002/glia.24642)
Supplement: Supplementary file 1 — Supplementary Figures: [file GLIA-73-422-s002.pdf]

# Figure S1

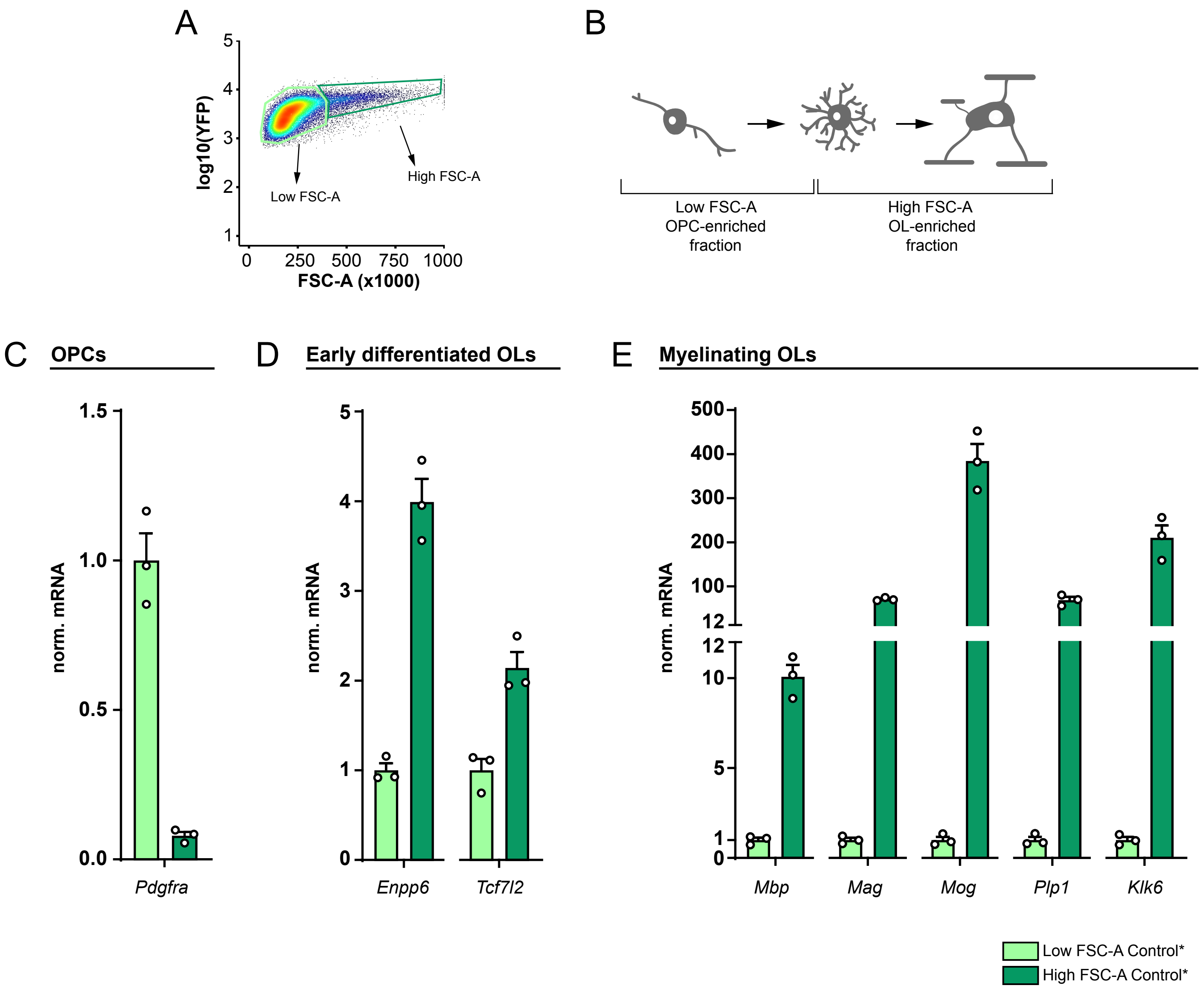


**Figure S1. Enrichment for oligodendrocyte precursor cells and differentiated oligodendrocytes in separate populations by FACS.**

**A)** Exemplary plot showing the gating strategy to enrich for oligodendrocyte precursor cells (Low FSC-A; OPCs) and differentiated oligodendrocytes (High FSC-A; OLs) sorted from SpCs at P10. Gating of YFP-positive cells (y-axis) and forward scatter area (FSC-A; x-axis) were used to select for recombined cells according to cell size. **B)** Schematic representation of the sorting populations obtained following the gating strategy depicted in (A). **C-E)** RNA of Control* cells acutely extracted according to the strategy depicted in (A) were submitted to RTqPCR analysis of genes expressed at different stages in the OL lineage and normalized to *Actinb*. The Low FSC-A mean value of each monitored gene was set to 1 (n = 3 mice per sample). **C)** *Pdgfra* was monitored as marker for OPCs. **D)** *Enpp6* and *Tcf7l2* were used as markers for early differentiated, not yet myelinating OLs. **E)** *Mbp*, *Mag*, *Mog*, *Plp1* and *Klk6* were analyzed as they are highly expressed in myelinating OLs (Marques et al., 2016; Xiao et al., 2016). Error bars indicate standard error of the mean (SEM).

# Figure S2

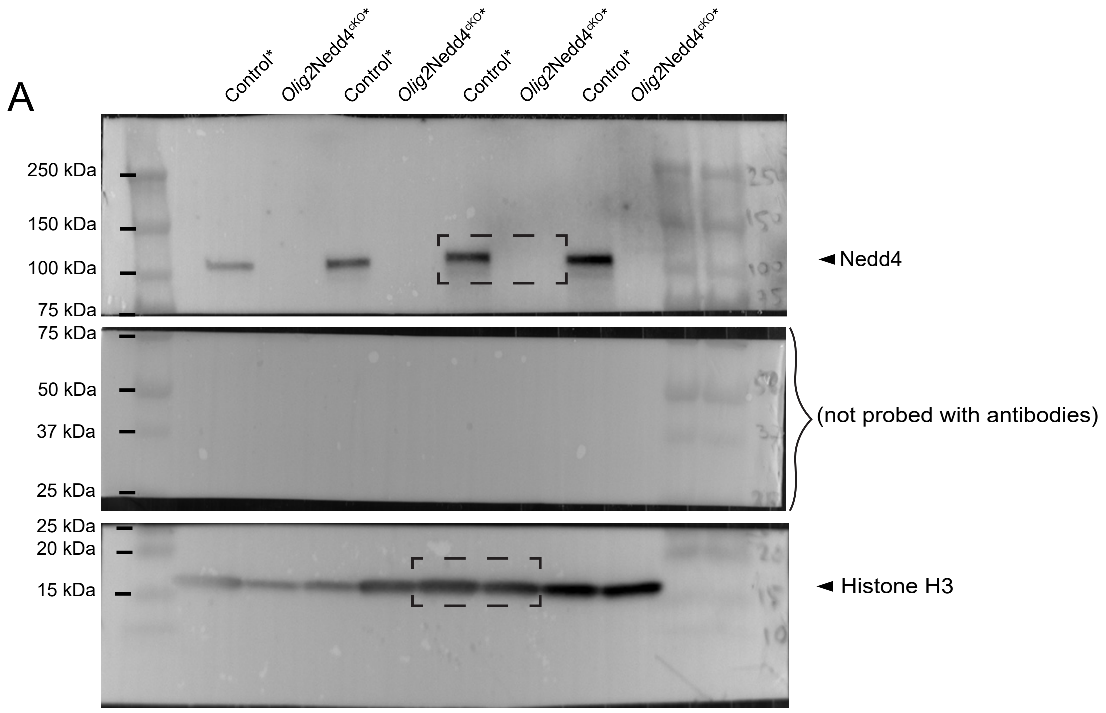


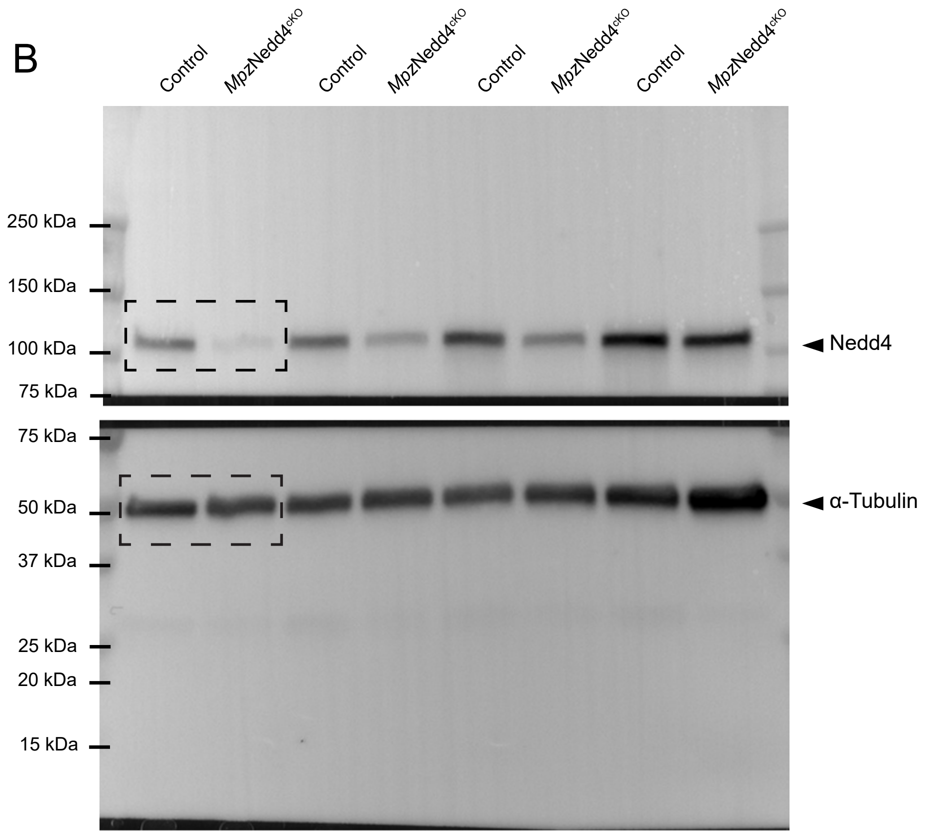


**Figure S2. Full-length blot images related to Figure 1 and Figure 6.**
**A)** Full images of western blots included in Figure 1B, showing the chemiluminescent signal overlaid with the respective brightfield image of the membrane. Dashed lines represent cropped images shown in Figure 1B as chemiluminescent signal only. Controls* and *Olig2*Nedd4$^{cKO*}$ samples are indicated above each corresponding lane. Membrane was cut at 75 kDa and 25 kDa prior to incubation with antibodies. **B)** Full images of western blots included in Figure 6B, showing the chemiluminescent signal overlaid with the respective brightfield image of the membrane. Dashed lines represent cropped images shown in Figure 6B as chemiluminescent signal only. Controls and *Mpz*Nedd4$^{cKO}$ samples are labelled above each corresponding lane. Membrane was cut at 75 kDa prior to incubation with antibodies. The molecular size shown next to the cropped bands refers to the apparent molecular weight estimated from Precision Plus Protein Standards (BioRad, Cat. #161–0373).

# Figure S3

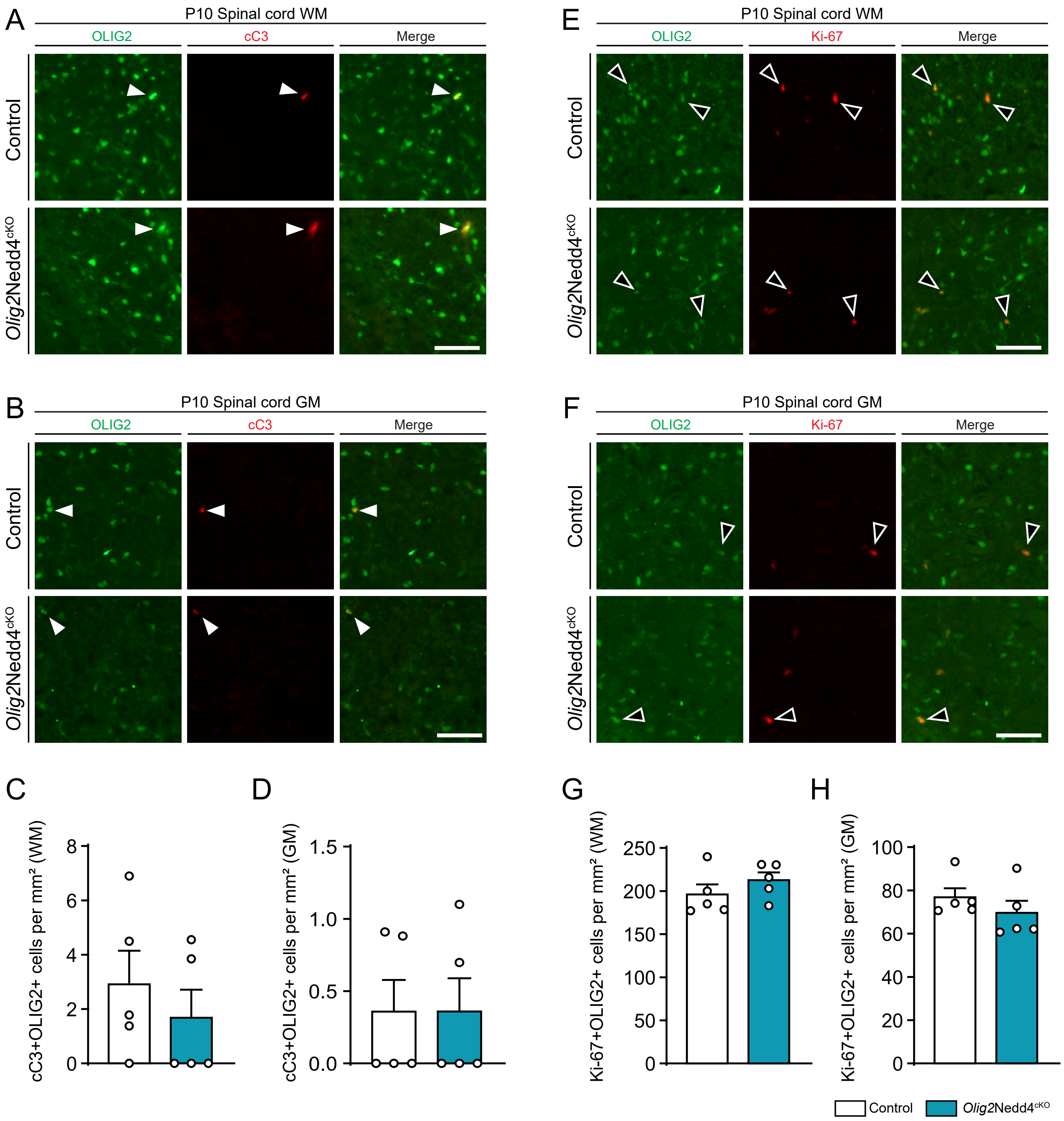


**Figure S3. No significant changes in apoptosis and proliferation among oligodendrocyte-lineage cells in P10 spinal cords from *Olig2*Nedd4$^{cKO}$ and Control mice.**

**A-B)** Exemplary immunostainings of cross-sections from *Olig2*Nedd4$^{cKO}$ and Control mice in SpC WM (A) and GM (B) at P10 labelled with cleaved caspase 3 (cC3) and OLIG2 antibodies. White arrowheads indicate examples of cC3+ OLIG2+ cells. Scale bar: 50 µm. **C)** Quantification of cC3+ OLIG2+ cells per mm$^2$ as shown in (A). **D)** Quantification of cC3+ OLIG2+ cells per mm$^2$ as shown in (B). **E-F)** Exemplary immunostainings of cross-sections from *Olig2*Nedd4$^{cKO}$ and Controls in SpC WM (E) and GM (F) at P10 labelled with Ki-67 and OLIG2 antibodies. Black arrowheads with white outline indicate examples of Ki-67+ OLIG2+ cells. Scale bar: 50 µm. **G)** Quantification of Ki-67+ OLIG2+ cells per mm$^2$ as shown in (E). **H)** Quantification of Ki-67+ OLIG2+ cells per mm$^2$ as shown in (F). **A-H)** n = 5 mice per genotype, two-tailed unpaired Student's t-test. Error bars indicate standard error of the mean (SEM).

# Figure S4

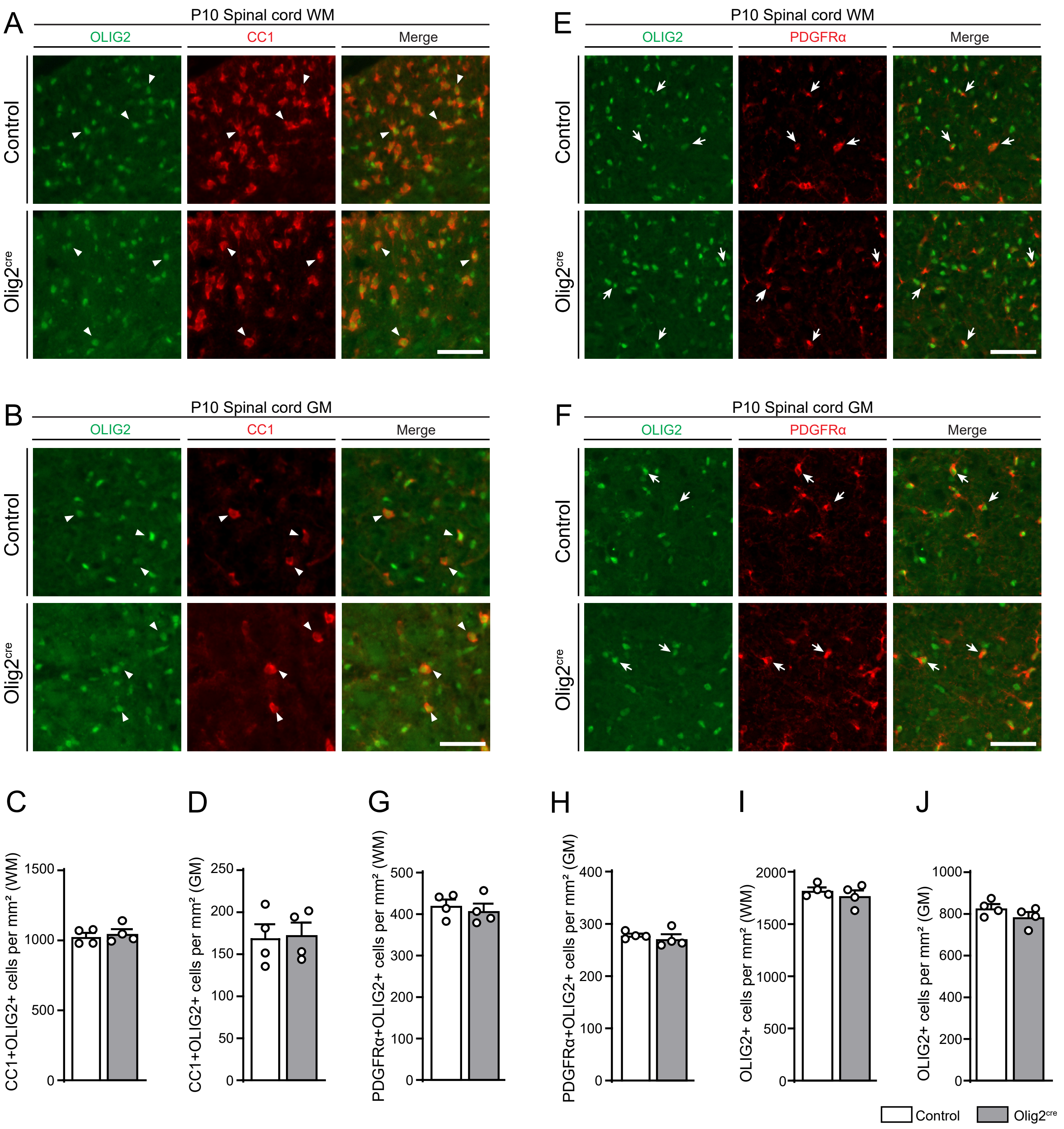


**Figure S4. The presence of only one *Olig2* functional allele in Olig2$^{cre}$ mice is not associated with significant alterations in the density of oligodendrocyte-lineage cells (OLIG2+), differentiated oligodendrocytes (CC1+ OLIG2+), or oligodendrocyte precursor cells (PDGFRα+ OLIG2+) compared to wildtype mice in P10 spinal cords.**

**A-B**) Representative immunostainings of cross-sections from P10 Olig2$^{cre}$ and Control (wildtype) mice in SpC WM (A) and GM (B) labelled with CC1 and OLIG2 antibodies. White arrowheads indicate examples of CC1+ OLIG2+ cells. Scale bar: 50 µm. **C)** Quantification of CC1+ OLIG2+ cells per mm$^2$ as shown in (A). **D)** Quantification of CC1+ OLIG2+ cells per mm$^2$ as shown in (B). **E-F)** Representative immunostainings of cross-sections from P10 Olig2$^{cre}$ and Control (wildtype) mice in SpC WM (E) and GM (F) labelled with PDGFRα and OLIG2 antibodies. White arrows indicate examples of PDGFRα+ OLIG2+ cells. Scale bar: 50 µm. **G)** Quantification of PDGFRα+ OLIG2+ cells per mm$^2$ as shown in (E). **H)** Quantification of PDGFRα+ OLIG2+ cells per mm$^2$ as shown in (F). **I)** Quantification of OLIG2+ cells per mm$^2$ as shown in (E). **J)** Quantification of OLIG2+ cells per mm$^2$ as shown in (F). **A-J)** n = 4 mice per genotype, two-tailed unpaired Student's t-test. Error bars indicate standard error of the mean (SEM).

# Figure S5

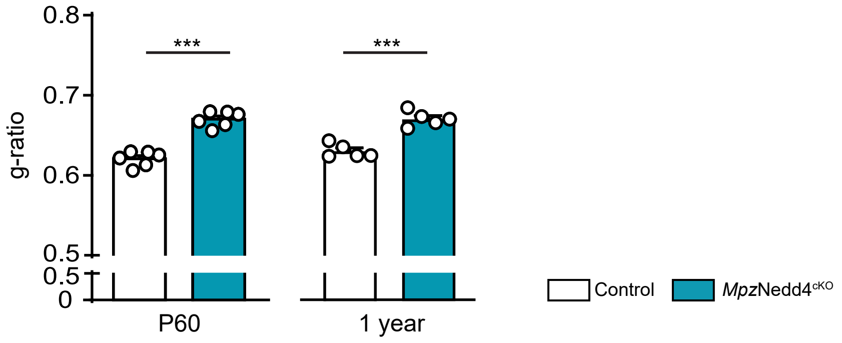


**Figure S5. Automated g-ratio quantification in sciatic nerves of *Mpz*Nedd4$^{cKO}$ and respective Controls.** Mean g-ratio values per mouse derived from automated quantification of myelinated axons in P60 and 1-year old *Mpz*Nedd4$^{cKO}$ compared to Control SNs (P60: n = 6 mice per genotype, 1483-2089 axons quantified per animal; 1 year: n = 5 mice per genotype, 1970-2302 axons quantified per animal; *** $p<0.001$; two-tailed unpaired Student's t-test). Error bars indicate standard error of the mean (SEM).

# Figure S6

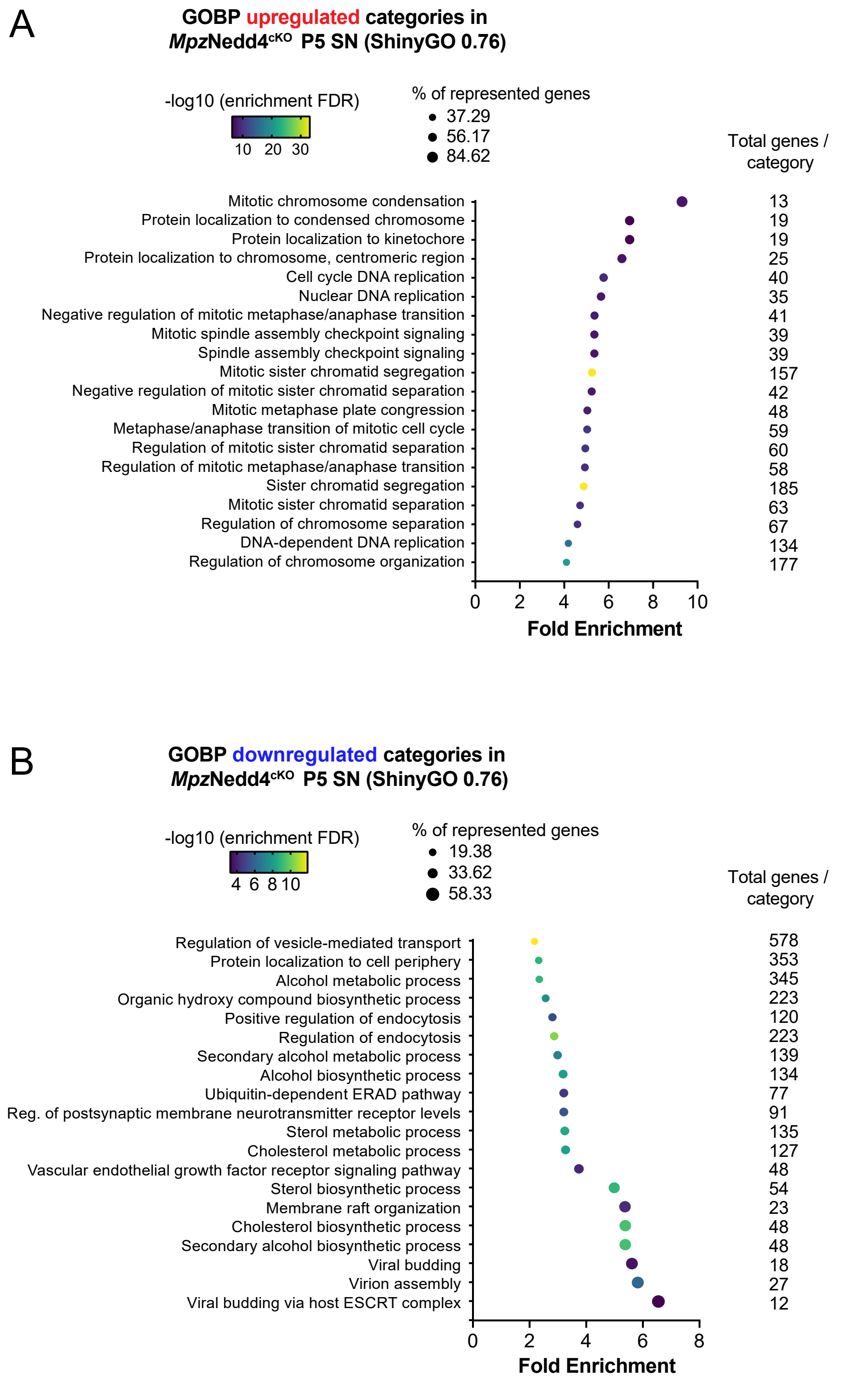


**Figure S6. Gene ontology of biological processes analysis with ShinyGo highlights similar categories as the analysis with EnrichR.**
**A)** ShinyGo GOBP analysis for significantly upregulated transcripts expressed by *Mpz*Nedd4$^{cKO}$ compared to Control SNs at P5 (FDR < 0.05; n = 4 mice per genotype). **B)** ShinyGO GOBP analysis for significantly downregulated transcripts expressed by *Mpz*Nedd4$^{cKO}$ compared to Control SNs at P5 (FDR < 0.05; n = 4 mice per genotype). Categories shown were sorted in ShinyGo by average ranks (FDR and fold change). The size of the dots relates to the percentage of genes represented in the dataset out of the indicated total genes in each category. The color of the dots relates to the heatmap showing the -log10 of enrichment FDR.
